# Glycoprotein 2 is a specific cell surface marker of human pancreatic progenitors

**DOI:** 10.1038/s41467-017-00561-0

**Published:** 2017-08-24

**Authors:** Kathryn F. Cogger, Ankit Sinha, Farida Sarangi, Emily C. McGaugh, Diane Saunders, Craig Dorrell, Salvador Mejia-Guerrero, Yasaman Aghazadeh, Jillian L. Rourke, Robert A. Screaton, Markus Grompe, Philip R. Streeter, Alvin C. Powers, Marcela Brissova, Thomas Kislinger, M. Cristina Nostro

**Affiliations:** 1 Toronto General Hospital Research Institute, University Health Network, Toronto, ON Canada M5G 1L7; 2 McEwen Centre for Regenerative Medicine, University Health Network, Toronto, ON Canada M5G 1L7; 3 Department of Medical Biophysics, University of Toronto, Toronto, ON Canada M5G 1L7; 4 Department of Physiology, University of Toronto, Toronto, ON Canada M5S 1A8; 5 Department of Molecular Physiology and Biophysics, Vanderbilt University, Nashville, TN 37232−0475 USA; 6 Oregon Stem Cell Center, Oregon Health and Science University, Portland, OR 97239-3098 USA; 7 Princess Margaret Cancer Centre, University Health Network, Toronto, ON Canada M5G 1L7; 8 Sunnybrook Research Institute, Toronto, ON Canada M4N 3M5; 9 Department of Biochemistry, University of Toronto, Toronto, ON Canada M5S 1A8; 10 VA Tennessee Valley Healthcare System, Nashville, TN 37212 USA; 11 Division of Diabetes, Endocrinology, and Metabolism, Department of Medicine, Vanderbilt University Medical Center, Nashville, TN 37232-0475 USA

## Abstract

PDX1^+^/NKX6-1^+^ pancreatic progenitors (PPs) give rise to endocrine cells both in vitro and in vivo. This cell population can be successfully differentiated from human pluripotent stem cells (hPSCs) and hold the potential to generate an unlimited supply of β cells for diabetes treatment. However, the efficiency of PP generation in vitro is highly variable, negatively impacting reproducibility and validation of in vitro and in vivo studies, and consequently, translation to the clinic. Here, we report the use of a proteomics approach to phenotypically characterize hPSC-derived PPs and distinguish these cells from non-PP populations during differentiation. Our analysis identifies the pancreatic secretory granule membrane major glycoprotein 2 (GP2) as a PP-specific cell surface marker. Remarkably, GP2 is co-expressed with NKX6-1 and PTF1A in human developing pancreata, indicating that it marks the multipotent pancreatic progenitors in vivo. Finally, we show that isolated hPSC-derived GP2^+^ cells generate β-like cells (C-PEPTIDE^+^/NKX6-1^+^) more efficiently compared to GP2^−^ and unsorted populations, underlining the potential therapeutic applications of GP2.

## Abstract (web summary)

Pancreatic progenitors (PPs) can be derived from human pluripotent stem cells in vitro but efficiency of differentiation varies, making it hard to sort for insulin-producing cells. Here, the authors use a proteomic approach to identify the secretory granule membrane glycoprotein 2 as a marker for PDX1+/NKX6-1+ PPs.

## Introduction

Exogenous insulin administration to individuals with type 1 diabetes (T1D) is a life-saving therapy, but does not mimic the fine-tuned blood glucose control achieved by insulin secretion from endogenous pancreatic islet β cells^1^. The success of whole pancreas and especially islet transplantation has provided compelling evidence that β cell-replacement therapy is a promising alternative treatment option for T1D, however the shortage of organ donors and required life-long immunosuppressive regimen limit their widespread use^1^. In contrast, hPSCs could provide an unlimited supply of insulin-producing cells, and together with immunoprotective or tolerogenic strategies could restore endogenous insulin secretion in patients with T1D and selected type 2 diabetics^2–5^. Differentiation protocols designed to mimic pancreatic organogenesis in vitro have been successfully used to generate hPSC-derived PPs. These PPs express PDX1 and NKX6-1, both markers of pancreatic progenitors, and have the potential to give rise to insulin-producing cells in vivo and in vitro^6–14^. While human embryonic stem cell (hESC)-derived PPs are currently being tested for safety in a clinical trial for patients with T1D (NCT 02239354), protocol reproducibility across hPSC lines has been challenging even within the same laboratory, with the percentage of hPSC-derived PPs ranging from 6–45%^7^ to 36–83%^9^.

Here, we compare the N-glycoproteome of undifferentiated hESCs, hESC-derived PP and hESC-derived polyhormonal (PH) cells to reveal novel surface markers that accurately distinguish hESC-derived PPs from contaminating cultures. Specifically, we identify the pancreatic secretory granule membrane major glycoprotein 2 (GP2) as a specific cell surface marker of hESC-derived PPs. We confirm expression of GP2 in sections of human developing pancreas, marking the NKX6-1^+^/PTF1A^+^ putative human multipotent progenitors. Furthermore, we demonstrate that GP2^+^ cells can be enriched using fluorescence- and magnetic-activated cell sorting techniques and that GP2^+^ purified populations have the potential to generate insulin-expressing mono-hormonal β-like cells in vitro.

Therefore, GP2 provides us with a tool to purify hESC-derived PPs as well as monitor the efficiency of in vitro pancreatic differentiation. Additionally, through the use of automated magnetic cell isolation, GP2 enrichment could easily be used for large-scale production of hESC-derived pancreatic progenitor cells for clinical and pharmaceutical purposes.

## Results

### HESC PP and PH N-glycoproteome profiling

**Fig. 1:**
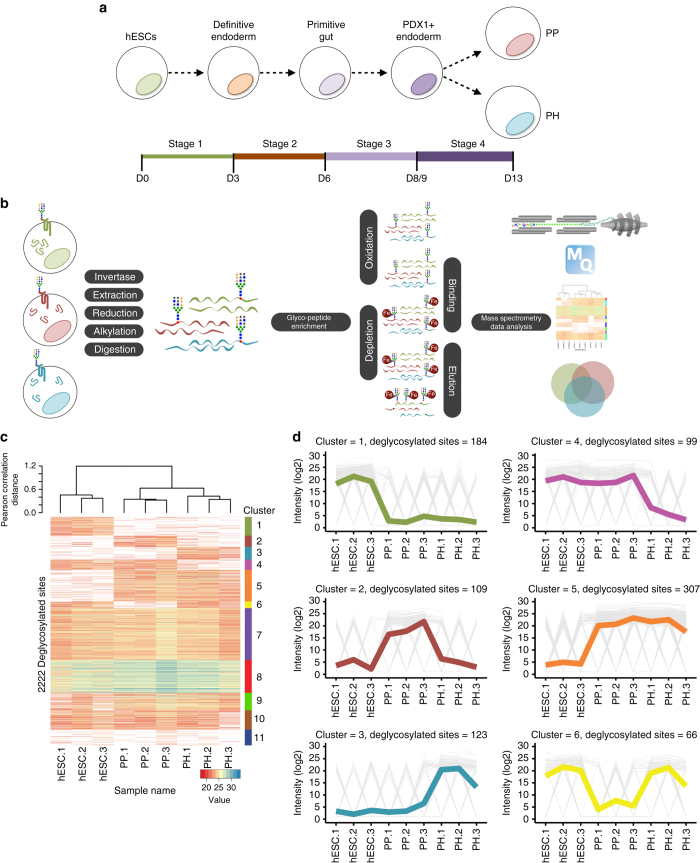
Membrane proteome analysis of undifferentiated hESCs and day 13 differentiated PP and PH cultures. **a** Schematic representation of the four stages of differentiation from human embryonic stem cells (hESCs) to pancreatic progenitor (PP) or polyhormonal (PH) cells. **b** Workflow of the strategy used for enriching *N*-linked glycosylated peptides for liquid chromatography—mass spectrometry. **c** Heatmap showing the 2222 identified deglycosylated sites, grouped into 11 clusters. Pearson correlation distances at the top show the relationship within the biological replicates, and between the different sample types. **d** *Line graphs* showing the intensity profiles of deglycosylated sites for the first six clusters. The *bold line* represents the mean intensity profile of all the deglycosylated sites in the respective cluster

To provide a safer cell population for therapeutic purposes and obviate the risk of contamination from undifferentiated hPSCs and/or other germ layer derivatives, we set out to identify specific cell surface markers that will allow for quantification and enrichment of hPSC-derived PPs. To this end, we quantitatively compared the glycoproteome of undifferentiated hESCs to hESC-derived PP cells. To increase specificity and identify cell surface markers exclusive to PPs, we differentiated hESCs toward two independent pancreatic populations: PPs and PH cells using two distinct differentiation protocols^15^ (Fig. 1a). Importantly, the PP population is primarily composed of PDX1^+^/NKX6-1^+^ cells (>80%) and shows no detectable expression of associated mature endocrine (*INS, GCG)* and pluripotency *(OCT4, SOX2)* genes (Supplementary Fig. 1a, b). Upon transplantation beneath the mouse kidney capsule, PPs are able to generate all lineages of the pancreas including β-cells^7–9, 12, 16^. In contrast, the PH differentiation protocol generates fewer NKX6-1^+^ cells (10.5% ± 1.1%) and a higher percentage of CPEP^+^/NKX6-1^−^ (10.0% ± 0.9%) and CPEP^+^/GCG^+^ (2.5% ± 0.3%) polyhormonal cells than the PP method (Supplementary Fig. 1b). PH cells also express significantly lower levels of *PDX1* and *NKX6-1* compared to PPs (Supplementary Fig. 1a) and do not generate β-cells in vivo^8, 13, 17^. Undifferentiated hESCs, along with the hESC-derived PP and PH cells were used for the selective enrichment of N-glycoproteins and compared by mass spectrometry (Fig. 1b). Characterization of the changes in the N-linked glycosylated proteome of hESC, PP and PH populations was performed using label-free shotgun proteomics of three independent replicates for each cell type. The analysis of the nine samples identified 2745 deamidation sites on asparagine residues, which mapped to 1043 protein groups (Supplementary Fig. 1c, d and Supplementary Data 1). Functional annotation revealed the greatest enrichment for receptor activity, receptor binding, receptors with catalytic functions, and transmembrane signal transduction (Supplementary Fig. 1e and Supplementary Data 2). Counts of identified proteins with receptor activity involved in signaling pathways highlights pathways such as axon guidance, cell adhesion molecules, PI3K, Ras and cytokine signaling (Supplementary Fig. 1f and Supplementary Data 3). Similar numbers of N-linked glycoproteins were identified in each cell population, of which 627 were shared among the three cell types and 35 were specific for the PPs (Supplementary Fig. 1g and Supplementary Data 4).

### N-glycoproteome comparison identifies proteins enriched in distinct cellular subsets

Pearson correlation-based unsupervised clustering of the nine samples revealed a higher correlation coefficient between PP and PH cells (0.53) rather than with hESC, which is supported by the observation of a large number of shared proteins between these two cell populations (Fig. 1c and Supplementary Fig. 1g). An internal control protein (yeast invertase—*SUC2*) confirmed data reproducibility and the absence of systematic bias (Supplementary Fig. 1h). K-means based unsupervised clustering was used to partition the 2222 deglycosylated sites into 11 distinct clusters based on their intensity profiles (Fig. 1c). Cluster 1, 2, and 3 contain deglycosylated sites that were uniquely detected in hESC, PP, or PH cells, respectively. The average intensity profiles for deglycosylated sites of the first six clusters are shown in Fig. 1d. The proteins assigned to each cluster are listed in Supplementary Data 5–10.

As expected, hESC reference markers such as ALPL, KDR, and SOX2 were all enriched in the hESC-specific cluster (Supplementary Data 5). Interestingly, KDR is also a known marker of mesodermal derivatives^18^, its protein and transcript profiles were validated by flow cytometry and qPCR, respectively (Supplementary Fig. 2a–c), confirming the absence of undifferentiated and mesodermal cells in the PP and PH cultures. Peptides for the epithelial and hESC marker EPCAM^18^, were detected in clusters 2 and 6 (Fig. 1d and Supplementary Data 6 and 10). These data were validated by flow cytometry and qPCR analysis (Supplementary Fig. 2a–c), which confirmed EPCAM expression in all populations and verified the epithelial nature of the PP and PH cells. Of the 126 proteins listed in cluster 1, 23 belonged to a PSC-restricted list identified by previous proteomic studies^19^, confirming the reliability of our approach (Supplementary Table 1).

### PP-specific cell surface markers

**Fig. 2:**
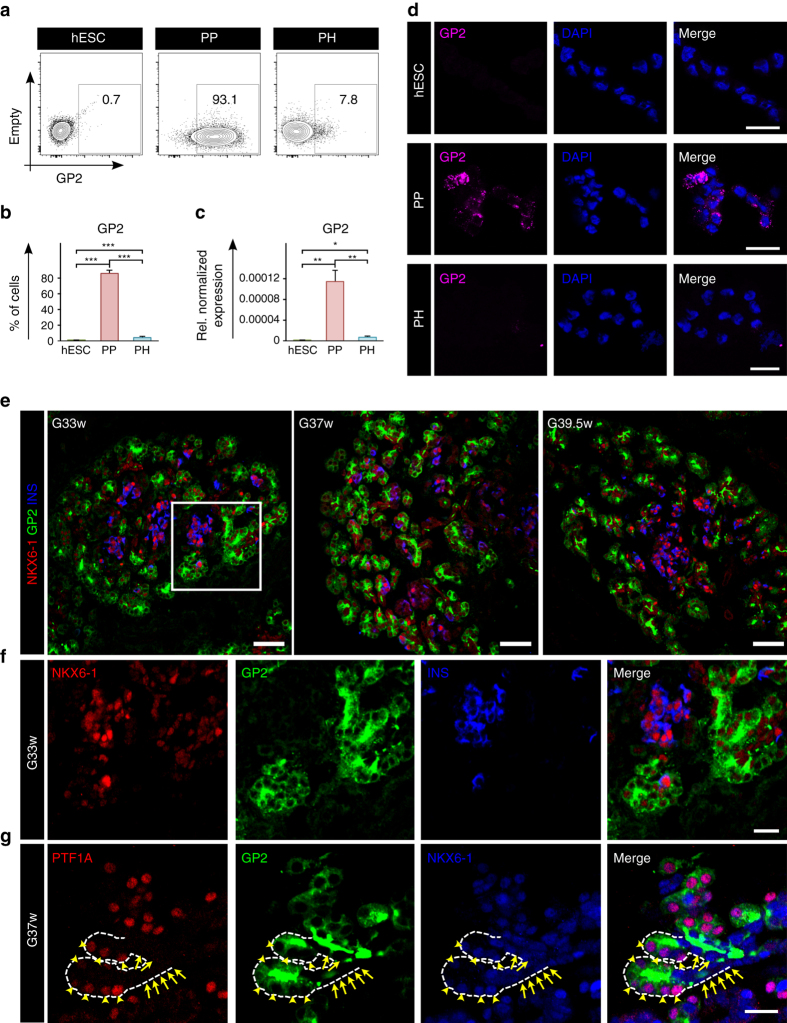
Validation of the PP marker GP2 by flow cytometry qPCR and immunocytochemistry. **a**,**b** Flow cytometry analyses of undifferentiated hESCs, and day 13 PP and PH cultures. Cells were stained with anti-GP2. *N* = 11 for hESC, *N* = 9 for PP and *N* = 8 for PH, *error bars* indicate s.e.m. ***p < 0.001,Student’s *t*-test. **c** qPCR analyses of *GP2* in undifferentiated hESCs, and day 13 PP and PH cultures. Expression levels normalized to *TBP*, and relative to adult pancreas (equal to 1, not shown). *N* = 3 for hESC and *N* = 4 for PP and PH, *error bars* indicate s.e.m. *p < 0.05, **p < 0.01, Student’s *t*-test. **d** GP2 immunostaining of undifferentiated hESCs, and day 13 PP and PH cultures. *Scale bar* represents 50 μM. **e**,**f** NKX6-1/GP2/INSULIN (INS) immunostaining of human pancreata at gestational weeks 33, 37, and 39.5, *scale bar* represents 100 μm. *White box* in *top panel* indicates area enlarged in *bottom panel*, *scale bar* represents 50 μm. **g** PTF1A/GP2/NKX6-1 immunostaining of human pancreata at gestational week 37. *White dotted line* indicates branching of the developing pancreas. *Yellow arrow heads* indicate PTF1A^+^/GP2^+^/NKX6-1^+^ cells at the tip of the branches. *Yellow arrows* indicate PTF1A^−^/GP2^−^/NKX6-1^+^ cells in the trunk of the branches. *Scale bar* represents 20 μm. Abbreviations: GP2, pancreatic secretory granule membrane major glycoprotein 2; NKX6-1, NK6 homeobox 1; PTF1A, pancreas specific transcription factor 1a; INS, insulin

With the goal to identify novel markers of the PP population, we next systematically selected proteins from clusters 2 and 5 (Fig. 1d and Supplementary Data 6 and 9) that presented the highest and most consistent changes in peptide intensity, and for which commercial antibodies were available for flow cytometry. Of the selected antibodies (Supplementary Table 2, Supplementary Fig. 2d), the pancreatic secretory granule membrane major glycoprotein 2 (GP2) was validated as an epitope that reliably and specifically marks the PPs, with over 85.0% ± 2.6% of cells expressing this marker by flow cytometry (Fig. 2a, b). *GP2* expression levels were also significantly higher in the PP compared to undifferentiated hESC and PH cells, as assessed by qPCR (Fig. 2c). We further tested the expression of GP2 in hESC-derived PPs by immunofluorescence analysis, demonstrating clear expression of GP2 in granule-like clusters in the PP cells, while the protein was undetectable in hESCs and PH cells (Fig. 2d).

### GP2 is a marker of putative PPs in human developing pancreas

GP2 expression in the human adult pancreas is restricted to the acinar compartment (http://www.proteinatlas.org/ENSG00000169347-GP2/tissue/pancreas#imid_17378936), which prompted us to investigate its expression profile at earlier developmental stages. To evaluate whether GP2 marks pancreatic progenitors, we performed immunofluorescence labeling for GP2, NKX6-1, PTF1A, and INS in human pancreatic tissues from neonatal donors born at 33, 37, and 39.5 weeks of gestation (G33w, G37w, and G39.5w, respectively). Remarkably, we uncovered an intriguing pattern of expression, where GP2^+^/NKX6-1^+^/INS^−^ cells are detected at the leading edge and INS^+^/NKX6-1^+^/GP2^−^ islet clusters are found in the center of the developing pancreatic lobes (Fig. 2e, f). To confirm the progenitor nature of the GP2^+^/NKX6-1^+^ cells, pancreatic sections were further co-labeled with antibodies directed to GP2, NKX6-1, and PTF1A; the latter is expressed by the nkx6-1^+^ multipotent progenitor cells residing in epithelial tips of the developing mouse pancreas^20, 21^. As shown in Fig. 2g, the cells located in the epithelial tip of the human pancreas at G37w do express PTF1A along with NKX6-1 and GP2 (Fig. 2g—arrow heads and Supplementary Fig. 2e). In contrast, the cells in the trunk express NKX6-1, but not PTF1A, showing a pattern similar to that observed in the mouse pancreas (Fig. 2g—arrows and Supplementary Fig. 2e). Notably, the trunk cells are also GP2 negative, indicating that this marker is specifically expressed in the putative multipotent progenitors during human pancreas development (Fig. 2g and Supplementary Fig. 2e).

### GP2 is a marker of hESC-derived PPs

**Fig. 3:**
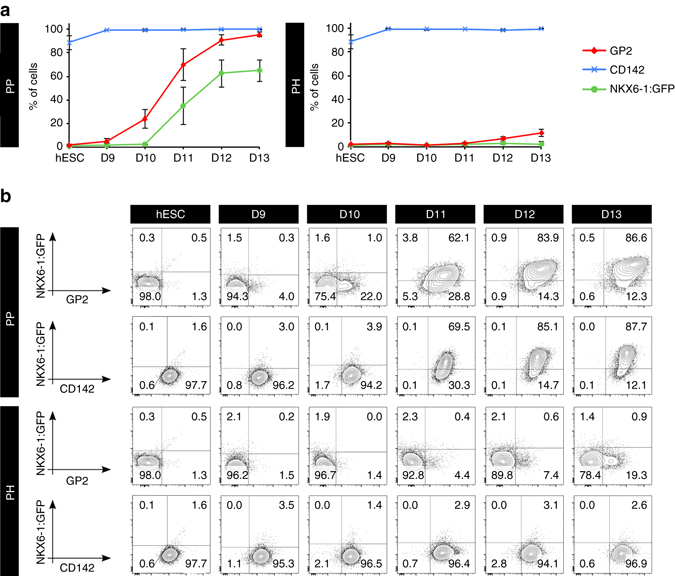
Time course analysis of GP2 expression in NKX6-1^*GFP/w*^ hESCs-derived PP and PH cultures. Flow cytometry analysis of undifferentiated NKX6-1^*GFP/w*^ hESCs and PP and PH cultures from day 9 to 13 of differentiation. Cells were stained with anti-GP2 or anti-CD142. **a** Average percentage of cells expressing GP2, CD142, or NKX6-1:GFP. *N* = 5 (hESC), *N* = 3 (D9-10), *N* = 4 (D12-13), *error bars* indicate s.e.m. **b** Representative flow cytometry plots. Abbreviations: D9, day 9; GP2, pancreatic secretory granule membrane major glycoprotein 2; CD142, tissue factor; NKX6-1, NK6 homeobox 1

**Fig. 4:**
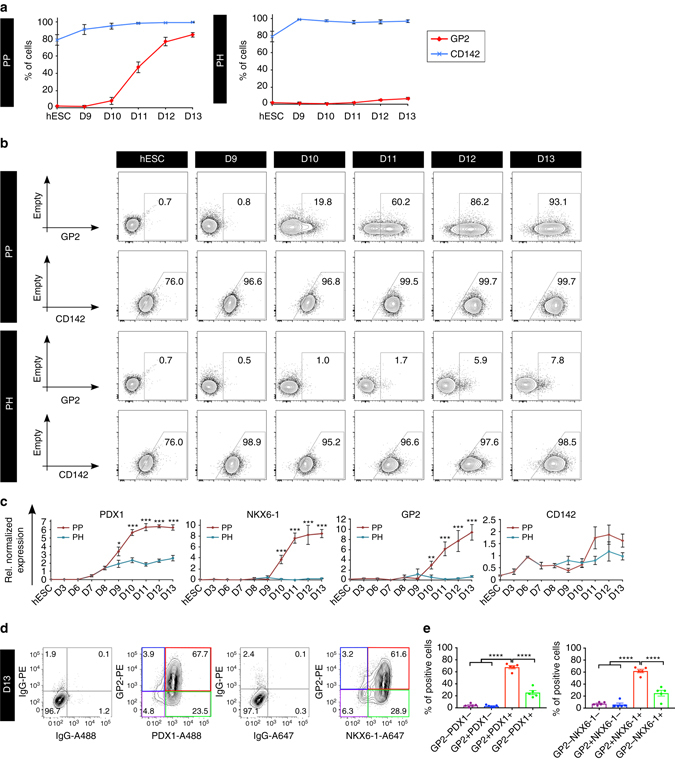
Time course analysis of GP2 expression in H1 hESCs-derived PP and PH cultures. Flow cytometry analysis of undifferentiated H1 hESCs and PP and PH cultures from day 9 to 13 of differentiation. Cells were stained with anti-GP2 or anti-CD142. **a** Average percentage of cells expressing GP2 *N* = 11 (hESC), *N* = 4 (D9-12), *N* = 9 (D13) or CD142 *N* = 5 (hESC), *N* = 3 (D9-112), *N* = 4 (D13). *Error bars* indicate s.e.m. **b** Representative flow cytometry plots. **c** qPCR analysis of *PDX1, NKX6-1, GP2*, and *CD142* from hESC to day 13. Expression levels normalized to *TBP*, and relative to adult pancreas (not shown). *GP2* relative normalized expression ×10^−5^. *N* = 3 for hESC, d3, d6, and *N* = 4 for d7-13, *error bars* indicate s.e.m. *p < 0.05, **p < 0.01, ***p < 0.001, Student’s *t*-test. **d** Representative flow cytometry analysis of day 13 cultures. Cells were stained for GP2 and PDX1 or NKX6-1 and IgG control antibodies. The different GP2/PDX1 and GP2/NKX6-1 fractions are identified by *purple*, *blue*, *red*, and *green boxes*. **e** Quantification of the GP2/PDX1 and GP2/NKX6-1 fractions, as measured by flow cytometry analysis at day 13, *N* = 5, *error bars* indicate s.e.m. ****p < 0.0001, One-way ANOVA. Abbreviations: D9, day 9; GP2, pancreatic secretory granule membrane major glycoprotein 2; CD142, tissue factor; NKX6-1, NK6 homeobox 1; OCT4, octamer-binding transcription factor 4; SOX17, SRY (sex determining region Y)-box 17; FOXA2, forkhead box A2; PDX1, pancreatic and duodenal homeobox 1; NGN3, neurogenin 3

Encouraged by this expression pattern, we set out to determine GP2 expression kinetic during hESC-directed differentiation using a reporter cell line that expresses GFP under the NKX6-1 promoter (NKX6-1^*GFP/w*^)^9^. Importantly, GP2 expression also correlated with the upregulation of NKX6-1-GFP during in vitro differentiation (Fig. 3a, b). GP2 was first detected at day 9 of differentiation, and was closely followed by NKX6-1-GFP expression at day 11. By day 13, all NKX6-1-GFP^+^ cells co-expressed GP2 (Fig. 3a, b). NKX6-1 was not detected in PH cultures, and less than 20% of the PH cells expressed GP2 at day 13, compared to 95.7% ± 1.9% in PP cultures (Fig. 3a, b). GP2 showed a similar expression pattern in the H1 cell line, with GP2 being first detected at day 10 (Fig. 4a, b). We next compared the expression of GP2 to CD142, a marker previously suggested to enrich pancreatic populations from PSC differentiation cultures^7^. CD142 was not detected in cluster 2 by the mass spectrometry analysis, but was instead found in cluster 7, comprising candidates expressed in all 3 cell populations analyzed. The CD142 antibody stained the undifferentiated hESCs as well as the entire PP and PH cell populations at all stages of differentiation. These patterns were observed in populations generated from both the NKX6-1^*GFP/w*^ and H1 cell lines (Fig. 3a, b and Fig. 4a, b), suggesting that this marker cannot enrich for NKX6-1^+^ cells, nor distinguish undifferentiated cells from pancreatic endoderm populations. qPCR data also showed no significant differences in the level of *CD142* expression between PP and PH differentiating cultures (Fig. 4c), further corroborating these results. In contrast, *GP2* expression is significantly upregulated from day 10 to 13 of differentiation in PP cells compared to PH cells and its profile is consistent with upregulation of *PDX1* and *NKX6-1* in the PP cultures (Fig. 4c). To further confirm that GP2 marks the human PP cells, we labeled H1-derived day 13 cultures with GP2, PDX1 and NKX6-1 antibodies. Compared to live staining, after fixation, the percentage of GP2^+^ cells decreased from 85% (Fig. 2b) to 70%, the majority of which were PDX1^+^ and NKX6-1^+^ (67 and 62%, respectively) (Fig. 4d, e), confirming the data obtained using the NKX6-1^*GFP/w*^ cell line (Fig. 3). Interestingly, not all NKX6-1^+^ cells are GP2^+^ (Fig. 4d, e), which is consistent with the existence of this population in the trunk region of the developing human pancreata (Fig. 2g). In addition to the anti-GP2 antibody, HPx1 and HPx2^22^, two antibodies raised against human exocrine pancreas, specifically labeled GP2^+^ cells (Supplementary Fig. 3a–g). Consistent with the kinetic profile of GP2 during PP differentiation, HPx1 and HPx2 co-stained the majority of NKX6-1-GFP-expressing cells in differentiating cultures (Supplementary Fig. 3h).

### FACS-enriched GP2^+^ cells generate β-like cells in vitro

**Fig. 5:**
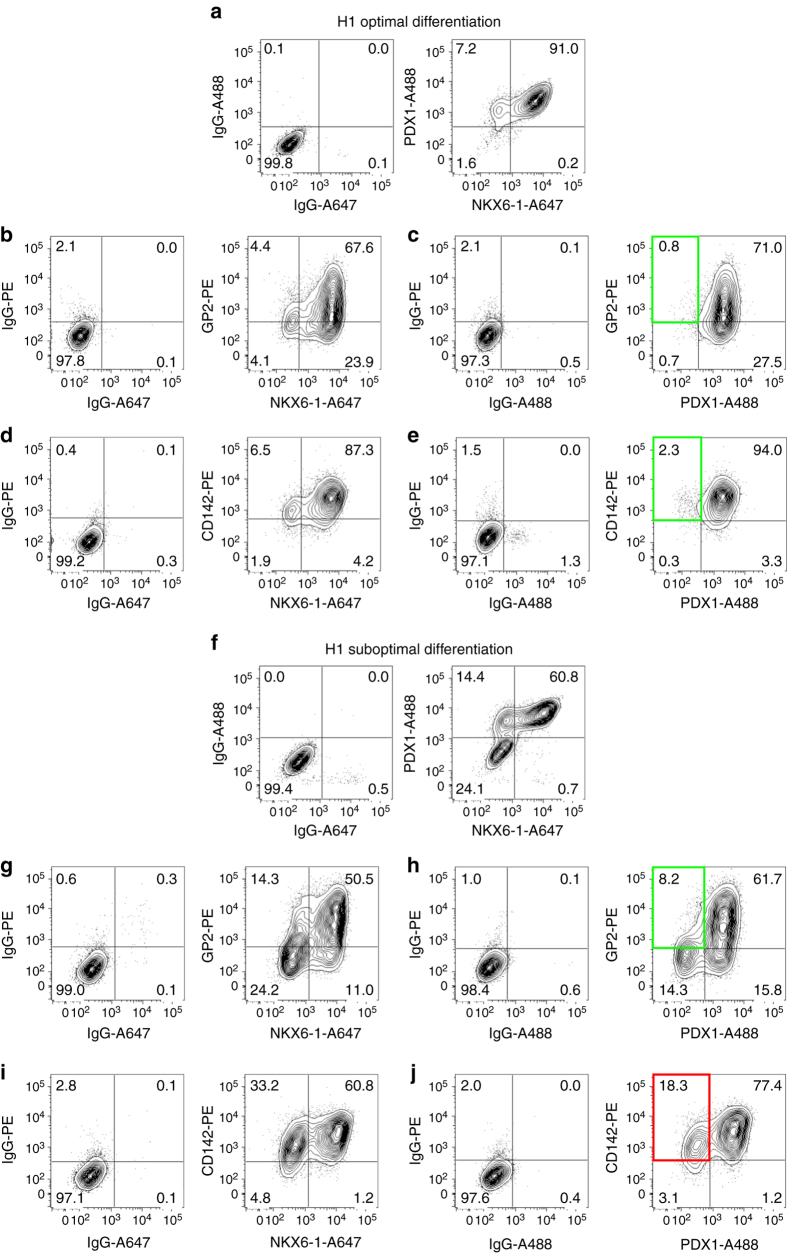
GP2 and CD142 comparative profiles. **a**–**j** Flow cytometry analyses of H1-derived day 13 PP cultures obtained using 10 mM nicotinamide (**a**–**e**, optimal differentiation) and 3.3 mM nicotinamide (**f**–**j**, suboptimal differentiation) during stage 4 of differentiation. Cells were stained with anti-GP2 and anti-CD142 and then fixed and stained for the intracellular markers PDX1 and NKX6-1. IgG controls are shown on the *left* of each panel. *Green boxes* highlight the low percentage of GP2+/PDX− cells (**c**–**h**), *red box* highlights the presence of CD142+PDX1− cells (**j**). Abbreviations: GP2, pancreatic secretory granule membrane major glycoprotein 2; CD142, tissue factor; NKX6-1, NK6 homeobox 1; PDX1, pancreatic, and duodenal homeobox 1

**Fig. 6:**
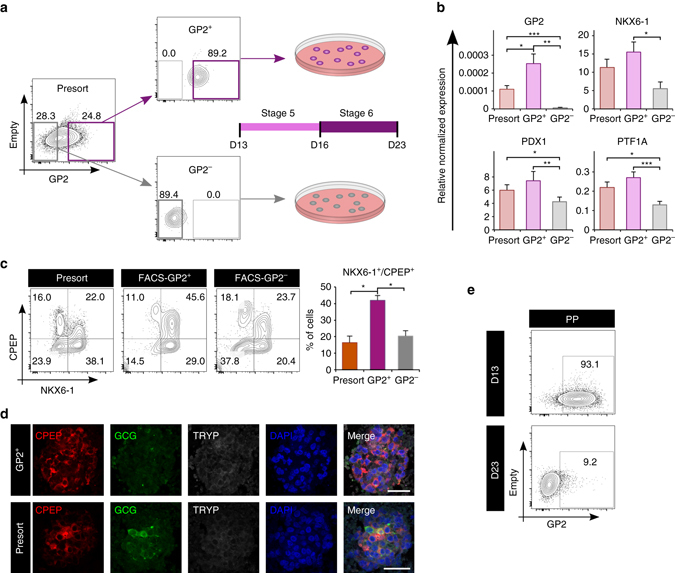
FAC-sorted GP2^+^ cells give rise to ‘β-like’ cells in vitro. **a** *Flow plots* showing the GP2 profile at day 13 of the unsorted (presort) and fluorescence-activated cell sorted H1 cells, which were then cultured to generate β-like cells up to day 23. **b** qPCR analysis of *GP2, NKX6-1, PDX1*, and *PTF1A*. Expression levels normalized to *TBP*, and relative to adult pancreas (equal to 1, not shown). *N* = 4, *error bars* indicate s.e.m. *p < 0.05, **p < 0.01, ***p < 0.001, Student’s *t*-test. **c** Representative flow cytometry plots of day 23 cultures from H1-derived unsorted (presort), GP2^+^ or GP2^−^ populations stained with anti-NKX6-1 and anti-C-PEPTIDE (CPEP) antibodies. The *bar graph* shows the average percentage of NKX6-1^+^/C-PEPTIDE^+^ cells. *N* = 5, *error bars* indicate s.e.m. *p < 0.05, One-way ANOVA. **d** C-PEPTIDE (CPEP)/GLUCAGON (GCG)/TRYPSIN (TRYP) immunostaining of GP2^+^ and H1-derived unsorted (presort) cultures at day 23 of differentiation. *Scale bar* represents 50 μm. **e** Flow cytometry plots showing GP2 expression at day 13 and day 23 of differentiation in unsorted (presort) H1 cells. Abbreviations: GP2, pancreatic secretory granule membrane major glycoprotein 2; D13, day 13; NKX6-1, NK6 homeobox 1; PDX1, pancreatic and duodenal homeobox 1; PTF1A, pancreas specific transcription factor 1a; CPEP, c-peptide; GCG, glucagon; TRYP, trypsin; INS, insulin

**Fig. 7:**
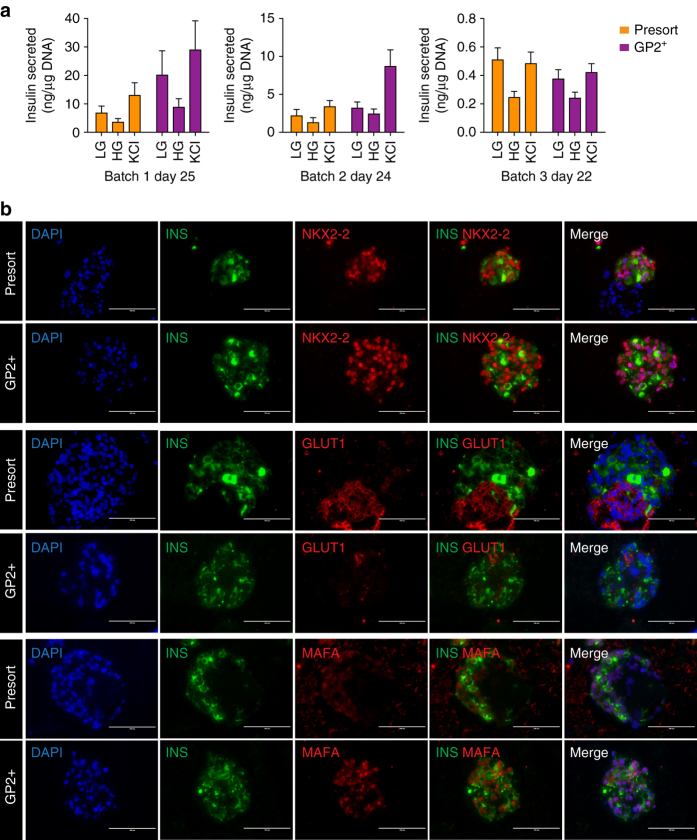
Functional and phenotypical characterization of presort- and GP2^+^ -derived aggregates. **a** Insulin secretion (ng insulin/μg DNA) from three different batches of aggregates generated from H1-derived presort and GP2^+^ cells at day 25, 24, and 22 of differentiation. *N* = 6 of 20–25 representative aggregates treated with low (2.8 mM) and high (16.7 mM) glucose and KCl (45 mM) for 45 min, *error bars* indicate s.e.m. **b** INSULIN (INS), NKX2-2, GLUT1 and MAFA immunostaining of unsorted (PRESORT) and GP2 + H1 cells at day 23 of differentiation. *Scale bar* represents 100 μm

To assess the use of GP2 as a cell surface marker to isolate pancreatic progenitor cells, we used fluorescence activated cell sorting (FACS) to isolate the GP2 positive and negative fractions at day 13 of differentiation using the H1 cell line. To ensure a sufficient pool of GP2^−^ cells, we reduced the amount of nicotinamide at stage 4 of differentiation^9^ to lower our differentiation efficiency and mimic the known variability that occurs with different PSC lines (Fig. 5). As shown in Fig. 5a, under optimal differentiation (high nicotinamide), the majority of cells expresses PDX1 and NKX6-1 at day 13 of differentiation and is positive for GP2 and CD142 (Fig. 5a–e). In contrast, in suboptimal conditions (low nicotinamide), the percentage of PDX1^+^/NKX6-1^+^ cells decreased and we could detect GP2^−^/PDX1^−^and GP2^−^/NKX6-1^−^ fractions. Conversely, CD142 binds to PDX1^−^ and NKX6-1^−^ cells, indicating that CD142 antibody cannot separate PDX1^+^ and NKX6-1^+^ from their negative fractions (Fig. 5f–j). These findings emphasize GP2 specificity compared to CD142 at marking hESC-derived PPs (Fig. 5b–e, g–j). Following a suboptimal differentiation, we generated day 13 cultures containing 36% and 35% GP2^+^ and NKX6-1^+^ cells, respectively (Supplementary Fig. 4a) and using FACS, we isolated the top 25% of GP2^+^ cells, and a similar percentage of the GP2^−^ population (Fig. 6a and Supplementary Fig. 4b, c). qRT-PCR analyses showed that *GP2* expression was significantly higher in the GP2^+^ sorted cells compared to the GP2^−^ and unsorted populations (Fig. 6b). Known markers of pancreatic progenitor cells *NKX6-1, PDX1, PTF1A, SOX9, C-MYC*, and *CPA1* ^20, 21, 23, 24^ were also expressed at significantly higher levels in the GP2^+^ compared to GP2^−^ cells (Fig. 6b and Supplementary Fig. 5a). To address whether the enriched GP2^+^ cells have an increased potential to generate β-like cells in vitro, the sorted and unsorted (presort) cells were cultured as aggregates in suspension, and assessed for their ability to give rise to NKX6-1^+^/CPEP^+^ cells^10, 11^. At day 23 of differentiation, cultures from GP2^+^ cells contained significantly more NKX6-1^+^/CPEP^+^ cells compared to GP2^−^ and unsorted cultures (Fig. 6c). Immunohistochemistry analysis confirmed the presence of PDX1^+^/NKX6-1^+^/CPEP^+^ cells and fewer GCG-expressing cells in the aggregates generated from the GP2^+^ population compared to presort, also confirming that the CPEP^+^ cells were monohormonal (Supplementary Fig. 5b and Fig. 6d). Notably, the percentage of GP2^+^ cells drastically declined from day 13 to day 23 (Fig. 6e), consistent with the absence of GP2 in endocrine cells (Fig. 2e, f and http://www.proteinatlas.org/ENSG00000169347-GP2/tissue/pancreas#imid_17378936). The day 23 GP2^+^- and presort-derived aggregates were also negative for pancreatic polypeptide, somatostatin, amylase, and trypsin, as assessed by immunofluorescence (Fig. 6d, and Supplementary Fig. 5c). To assess the functionality of the insulin-producing cells generated from the different fractions, we performed a glucose-stimulated insulin secretion assay at day 22, 24, and 25 of differentiation in three different batches of cells and showed no increase in insulin secretion in response to high glucose concentration, indicating that these cells were not glucose responsive (Fig. 7a). These data prompted us to assess the expression of additional β-cell markers NKX2-2, MAFA, and GLUT1. Immunofluorescence analysis confirmed co-expression of NKX2-2 and MAFA with the majority of INS^+^ cells in both presort- and GP2^+^-derived aggregates. Remarkably, GLUT1, the glucose transporter required for glucose sensing in humans, was not co-expressed by the INS^+^ cells, indicating lack of complete maturation and consistent with the absence of glucose response (Fig. 7b).

### HESC and hiPSC-derived GP2^+^ cells can be enriched using MACS

**Fig. 8:**
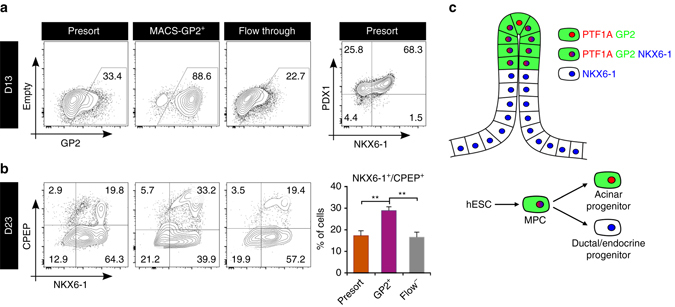
MAC-sorted GP2^+^ cells give rise to ‘β-like’ cells in vitro. **a** Flow plots showing the GP2 profile at day 13 of differentiation of H9 cells. Cells were analyzed either before MACS sorting (presort), after GP2 enrichment from a positive selection column (GP2^+^) or in the flow through from a depletion column. *Top right plot* shows NKX6-1 and PDX1 expression by flow cytometry in day 13 unsorted (presort) H9 cells. **b** Following MACS sorting for GP2 at day 13, cells were cultured generate β-like cells up to day 23. Representative flow cytometry plots of NKX6-1 and C-PEPTIDE (CPEP) expression at day 23 of differentiation from either unsorted (PRESORT), enriched for GP2 using a MACS positive selection column (GP2^+^) or in the flow through cell population (Flow-). The *bar graph* shows the average percentage of NKX6-1^+^/C-PEPTIDE^+^ cells at Day 23. *N* = 4, *error bars* indicate s.e.m. **p < 0.01, One-way ANOVA. **c** Model depicting the in vivo and in vitro equivalent of the human multipotent pancreatic progenitor (MPC) expressing PTF1A/GP2/NKX6-1. The MPC residing at the tip of the developing human pancreas has the potential to develop into acinar (PTF1A^+^/GP2^+^) and ductal/endocrine (NKX6-1^+^/GP2^−^) progenitors. Abbreviations: GP2, pancreatic secretory granule membrane major glycoprotein 2; D13, day 13; NKX6-1, NK6 homeobox 1; PDX1, pancreatic and duodenal homeobox 1; PTF1A, pancreas specific transcription factor 1a

Overall, our study identifies GP2 as a specific marker of human pancreatic progenitors and describes an efficient strategy to purify these precursors from hPSCs. However, FACS is time-consuming, costly and would not allow for feasible cell purification from large-scale cultures for clinical/pharmaceutical purposes. Magnetic-activated cell sorting (MACS) provides a system to process large numbers of cells in a shorter amount of time compared to FACS and most importantly can be automated, which would simplify the process to move into GMP-compliancy. To validate the use of GP2 for translational purposes and to demonstrate efficient labeling of the population of interest, we utilized H9, a cell line that could be used for clinical application (cGMP line distributed by WiCell) and that had previously shown substandard differentiation to the pancreatic lineage^9^. Indeed, day 13 H9-derived cultures contained a lower percentage of GP2^+^ and NKX6-1^+^/PDX1^+^ cells compared to H1-derived cultures (Fig. 8a and Supplementary Fig. 1a). In order to characterize the H9-derived GP2^+^ cells, we co-stained day 13 cultures for GP2, NKX6-1 and PDX1 and demonstrated that the GP2^+^ fraction is almost exclusively PDX1^+^ and over 90% PDX1^+^NKX6-1^+^ (Supplementary Fig. 6a). H9-derived GP2^+^ cells were successfully isolated using MACS and cultured alongside cells from both the presort and flow through fractions. As expected the H9-derived GP2^+^-MACS-sorted population was enriched in GP2^+^ and PDX^+^NKX6-1^+^ cells, while the flow through maintained similar percentage of GP2^+^ and PDX1^+^/NKX6-1^+^ as the presort (Supplementary Fig. 6). Similarly to the H1-fluorescence-activated cell sorted GP2^+^ population, the magnetic-activated sorted GP2^+^ cells gave rise to significantly more NKX6-1^+^/CPEP^+^ cells compared to flow through and unsorted populations (Fig. 8b). To verify that this method can be applied to additional cell lines, we isolated GP2^+^ cells using MACS from BJ-iPSC1, a human induced pluripotent stem cell line (hiPSC). Day 13 BJ-iPSC1-derived cultures comprised of 44% PDX1^+^/NKX6-1^+^ and 76% GP2^+^ cells (Supplementary Fig. 7a, b). BJ-iPSC1-derived GP2^+^ cells were successfully isolated using MACS and cultured alongside cells from both the presort and flow through fractions. Similar to the H1 and H9 sorted GP2^+^ populations, the magnetic-activated cells sorted GP2^+^ cells gave rise to significantly more NKX6-1^+^/CPEP^+^ cells compared to flow through and unsorted populations (Supplementary Fig. 7c, d), demonstrating the feasibility of this approach.

## Discussion

GP2 is the most abundant membrane protein found in zymogen granules of the pancreatic acinar cells^25^, and is secreted along with digestive enzymes into the small intestine where it acts as an immunomodulator^26^. GP2 knockout mice develop normally and do not have metabolic impairments, suggesting that this protein is not required for normal pancreas organogenesis nor function^27^. Nevertheless, driven by the proteomic results showing that GP2 is highly enriched in the hESC-derived pancreatic progenitor cells, we set out to investigate the expression of GP2 in the human postnatal pancreas and revealed the existence of a subset of cells co-expressing PTF1A and NKX6-1 that localize to the epithelial tips in the human pancreas at birth (Fig. 2g). Our findings strongly suggest that in vivo, GP2^+^/PTF1A^+^/NKX6-1^+^ cells may correspond to the multipotent pancreatic progenitor cells that have been described in several alternative model organisms^20, 21, 23, 24^ (Fig. 8c). To start characterizing the differentiation potential of such cells, we confirmed the existence of NKX6-1^+^/GP2^+^ cells in vitro in differentiating cultures of hESC and importantly, we demonstrated that FACS and MACS-purified hPSC-derived GP2^+^ cells harbor the potential to efficiently generate β-like cells in vitro, albeit lacking GLUT1 and not glucose responsive (Figs. 3–8). These findings are consistent with mouse studies^20, 21^, but suggest that contrary to the murine multipotent pancreatic progenitor, which exists only during embryonic life, the human multipotent pancreatic progenitor may persist postnatally. Lineage tracing studies in the mouse have clearly demonstrated the ability of the MPC to give rise to acinar, ductal and endocrine cells^21, 23^. Here, we speculate that the NKX6-1^+^/PTF1A^+^/GP2^+^ cells represent the human MPCs and that a similar cell population can be generated in vitro from hPSCs (Fig. 8c). Future studies will have to define whether the hESC-derived GP2^+^ cells have the ability to generate all the lineages of the pancreas at a clonal level.

Using RNA-sequencing, a recent study identified 500 MPC-specific transcripts^28^. When we mapped the 500 MPC-specific transcripts to their gene products using the UniProt database, we were able to map 499 proteins, of which 174 are N-glycosylated (based on annotation). Out of these 174 proteins, 33 were also enriched in clusters 2, 4, and 5, confirming the reliability of the mass-spectrometry analysis and the use of hESC-directed differentiation to model human development (Supplementary Data 11). Surprisingly, GP2 was not among the MPC-enriched transcripts. While the percentage of GP2^+^ cells is very high in the hESC-derived PPs (Fig. 2a, b), *GP2* expression at day 13 of differentiation in the GP2^+^ fraction is ~3000-fold lower compared to adult pancreas (Fig. 6b). In comparison, *PTF1A* (a known marker of MPC), which has been correctly identified by the RNAseq analysis performed by Cebola et al.^28^, is expressed fourfold less in hESC-derived GP2^+^ cells compared to adult pancreas (Fig. 6b), suggesting that *GP2* expression level in the MPC might be below detection for RNA-sequencing analysis. This is consistent with studies demonstrating poor correlation between cell surface protein levels and transcript copy number^29^ and reinforces the advantage of proteomics for immunophenotyping. With respect to the potential clinical applications of ES-cell-derived PPs, the identification of GP2 as a PP marker provides us with an unprecedented tool to isolate and quantify hPSC-derived PPs. Whether enriching for GP2 delivers a safer cell population for clinical purposes remains to be demonstrated by transplantation studies. Nevertheless, the use of the GP2 antibody coupled with MACS could provide a method that can be easily translated to clinical practices for large-scale production of enriched human pancreatic progenitor cells that could be used directly in clinical trials or to enable the in vitro production of human β cells in GMP conditions. Furthermore, GP2 expression may form part of a validation step in the manufacturing pipeline for future PP or PP-derived cellular therapies.

During the revision of this manuscript GP2 was identified by microarray analysis as a specific cell surface marker for isolating pancreatic endoderm cells (PECs) from differentiated hESCs and human fetal pancreas^30^. This complementary approach adds strength and reproducibility to the identification of GP2 as a novel marker of the human pancreatic progenitors cells and importantly demonstrates that by using a media containing forskolin, ALK5i, Noggin, and nicotinamide, GP2^+^ cells can give rise to insulin-secreting β cells, as measured by a static glucose-stimulated insulin secretion assay. Further studies will be required to evaluate whether GP2^+^ cells cultured in forskolin, ALK5i, Noggin, and nicotinamide express GLUT1 and secrete insulin in a perifusion assay.

## Methods

### Culture and differentiation of hESCs

H1 and H9 hESCs were obtained from WiCell; NKX6-1^*GFP/w*^ hESCs were provided by Drs. Stanley and Elefanty^9^. BJ-iPSC1 was provided by Drs. Araki and Neel^31^. Undifferentiated hESCs tested negative for mycoplasma and were maintained as previously described^32^. Differentiation was initiated when the hESC cultures reached 70−80% confluence. Monolayer cultures were treated with RPMI (Gibco) containing 100 ng/ml hActivin A (R&D Systems) and 2 μM CHIR990210 (Tocris) for one day (d0-d1). They were then cultured for 2 days in RPMI media containing 100 ng/ml hActivin A and 5 ng/ml hbFGF (R&D Systems) (d1–d3). This completed stage 1 of differentiation. During stage 2, cells were cultured in RPMI with 1% vol/vol B27 supplement (without vitamin A) (Life Technologies), 50 ng/ml hFGF10 (R&D Systems), 0.75 μM dorsomorphin (Sigma), and 3 ng/ml mWnt3a (R&D Systems) (d3−d6). On day 6 of differentiation, cultures were transferred to stage 3 media, consisting of DMEM (Gibco) with 1% vol/vol B27 supplement, 50 μg/ml ascorbic acid (Sigma), 50 ng/ml hNOGGIN (R&D Systems), 50 ng/ml hFGF10, 0.25 μM SANT-1 (Tocris), and 2 μM all-trans RA (Sigma), and cultured for 2–3 days. During stage 4, the media was changed to DMEM containing 1% vol/vol B27 supplement, 50 μg/ml ascorbic acid and 50 ng/ml hNOGGIN, and supplemented with either 100 ng/ml hEGF (R&D Systems) and 10 mM nicotinamide (Sigma), to direct cells toward the PP lineage (d8–d13), or 6 μM SB431542 (Sigma) to generate PH cells (d9–d13). H1 cells for FACS sorting received media supplemented with 100 ng/ml hEGF and 3.3 mM nicotinamide at stage 4 to obtain a GP2-low expressing population. H9 cells received stage 3 media for 3 days, and received media supplemented with 50 ng/ml hEGF and 10 mM nicotinamide at stage 4, as previously described^9^. BJ-iPSC1 cells received stage 3 media for 1 day, and received media supplemented with 100 ng/ml hEGF and 10 mM nicotinamide at stage 4, as previously described^9^.

For stage 5 and 6, single cells obtained after FAC- and MAC-sorting were cultured in suspension at 2 × 10^6^ cells/ml in low-adherent tissue culture plates in MCDB131 media (Gibco) containing 1 μM T3 (Sigma), 1.5 g/L NaHCO_3_ (Gibco), 1% vol/vol l-glutamine (GE Healthcare), 1% vol/vol B27 supplement (Gibco), 15 mM D-(+)-glucose (Sigma), 10 μg/ml heparin (Sigma), 0.25 μM SANT-1 (Tocris), 10 μM RepSox (Tocris), 100 nM LDN193189 (Cayman), 10 μM ZnSO_4_ (Sigma), 0.05 μM all-trans RA (Sigma), and 10 μM Y27632 (Tocris) (d13–d16, stage 5). After 72 h, the media was changed to MCDB131 containing 1 μM T3, 1.5 g/L NaHCO_3_, 1% vol/vol l-glutamine, 1% vol/vol B27 supplement, 15 mM D-(+)-Glucose, 10 μg/ml Heparin, 10 μM RepSox, 100 nM LDN193189, 10 μM ZnSO_4_, and 100 nM DBZ (Tocris) (d16–d23, stage 6), media was replenished at day 19. Aggregates were collected at day 23, dissociated with trypsin for flow cytometry or fixed in 1.6% PFA for immunohistochemistry analysis. Stage 5 and stage 6 media components are based on the previously described protocols^10, 11^.

### Digestion of proteins for mass spectrometry-based proteomics

Obtained cell pellets were re-suspended in 50% (vol/vol) 2,2,2-trifluoroethanol in phosphate buffer saline (pH 7.4) and 10 pmol of invertase protein (*SUC2*, a yeast glycoprotein, Sigma-Aldrich; UniProt accession—P00724) was spiked-in each sample as an internal method control. Cell lysis was induced through pulse sonication. Subsequently, lysates were incubated at 60 °C for 2 h with brief agitation every 30 min. The cysteines in the protein lysates were reduced through the addition of 5 mM dithiothreitol and incubated at 60 °C for 30 min. The lysates were cooled prior to alkylation of reduced cysteines, which was performed with 25 mM iodoacetamide at room temperature for 30 min in the dark. Subsequently, all samples were diluted 1:5 (v/v) using 100 mM ammonium bicarbonate (pH 8.0). Mass-spectrometry grade trypsin (trypsin:protein ratio 1:50, Promega) was added and digestion was performed at 37 °C for 16 h. Peptides were desalted using C18 MacroSpin Columns (Nest Group) and lyophilized using a vacuum concentrator.

### Enrichment of N-glycosylated peptides

Solid Phase extraction of N-glycosylated Peptides (SPEG) strategy was used for enriching N-glycosylated peptides from a pool of peptides, as shown in Fig. 1b ^33^. Briefly, lyophilized peptides were re-suspended in 100 mM sodium acetate/150 mM sodium chloride at pH 5.5. Glycans present on peptides were oxidized by adding 10 mM sodium meta-periodate and incubated at room temperature for 30 min in the dark. Peptides were desalted, lyophilized and solubilized in the sodium acetate buffer. Peptides with oxidized-glycans were captured on hydrazide labeled magnetic beads (glycoprotein:bead ratio 1:1, Chemicell) for 16 h with constant rotation. The supernatant containing unbound peptides was aspirated, and the beads were washed with 1.5 M sodium chloride, water, methanol, acetonitrile, and 100 mM ammonium bicarbonate at pH 8.0. Bound peptides were eluted from the beads using PNGaseF (Roche), and incubated at 37 °C for 16 h. The supernatant containing the deglycosylated peptides was desalted, lyophilized and measured for peptide concentration using the Thermo Scientific Nanodrop 2000 spectrophotometer.

### Mass spectrometry and peptide/protein identification

A total of 1.5 µg of deglycosylated peptides were injected for each LC-MS/MS analysis^34^. Briefly, peptides were separated using reverse-phase chromatography with a 4 h gradient. Chromatography was performed using a 50 cm column with a flow rate of 250 nL/min using the Thermo Scientific EasyLC1000 nano-liquid-chromatography system. QExactive tandem mass spectrometry was used for acquiring MS/MS data while operating in a data dependent mode. The acquired raw-files were analyzed with MaxQuant (version: 1.5.0.0) using UniProt complete human proteome protein sequence database (version: 2012-07-19, number of sequences: 20,232)^35^. Searches were performed with a maximum of two missed cleavages, carbamidomethylation of cysteine was specified as a fixed modification, and oxidation of methionine and deamidation of asparagine to aspartic acid were specified as variable modifications. False discovery of peptides was controlled using a target-decoy approach based on reverted sequences, and the false discovery rate was defined as 1% at site, peptide, and protein levels.

### Mass spectrometry data analysis

All analysis was based on the data present in the Maxquant output file named Asn-_AspSites.txt and performed using R. All deamidated sites containing either SER or THR at the +2 site of the deamidated-ASN were carried forward for analysis, and were defined as deglycosylated sites. For each cell type, a deglycosylated site was considered to be present if it was quantified in at least two replicates. All gene ontology, pathway and protein keyword analysis was performed using ProteinCenter (Thermo Scientific). Unsupervised clustering of samples was performed using Pearson correlation, and K-means unsupervised learning algorithm was used for clustering deglycosylated sites into 11 clusters (determined using scree plot of sum of squared error) based on their intensity profile in the three biological samples.

### Flow cytometry and fluorescence-activated cell sorting (FACS)

Cells were dissociated from the monolayer using either TrypLE Express (Gibco) or StemPro Accutase (Gibco) and incubated at 37 °C to generate a single-cell suspension. Live cells were incubated for 20 min at 4 °C with primary antibodies in 1X PBS/10% fetal bovine serum (FBS; Wisent Inc.) (FACS buffer). After washing twice with FACS buffer, samples labeled with unconjugated primary antibodies were incubated for an additional 20 min at 4 °C with secondary antibodies in FACS buffer.

For FACS, cells were processed as above, and re-suspended in 1X PBS/0.1% FBS. They were sorted using the BD AriaII-RITT or AriaIII Fusion cell sorters.

For all intracellular staining, except PDX1, cells were fixed in 1.6% paraformaldehyde (PFA) for 24 h at 4 °C. Samples were then washed twice in FACS buffer, and incubated overnight at 4 °C with primary antibodies in 1X PBS containing 5 mg/ml saponin (Sigma) (saponin). After two washes in FACS buffer, the cells were then incubated with secondary antibodies in saponin for 30–45 min at room temperature. For PDX1 intracellular staining, cells were fixed in BD Bioscience cytofix/cytoperm buffer (Cat.# 554722) for 24 h at 4 °C. They were then washed twice in 1X BD Bioscience Perm/Wash (Cat.# 554723) and incubated with anti-PDX1 primary antibody (1/100, R&D Systems AF2419) in BD Bioscience Perm/Wash, for 1 h at room temperature. Following 2 washes in the BD Bioscience Perm/Wash, a donkey α goat AF647 or donkey α goat AF488 secondary antibody (Jackson ImmunoResearch Laboratories Inc. 705-606-147 or 705-546-147) diluted 1/400 in Perm/Wash was applied, and the cells incubated again for 1 h at room temperature.

For the triple staining GP2-PDX1-NKX6-1 and CD142-PDX1-NKX6-1, live cells were incubated for 20 min at 4 °C with an anti-GP2 antibody or anti-CD142 antibody in 1X PBS/10% fetal bovine serum (FBS; Wisent Inc.) (FACS buffer). After washing once with FACS buffer, the live cells were incubated for an additional 20 min at 4 °C with an anti-mouse PE-conjugated secondary antibody in FACS buffer. The cells were fixed in BD Bioscience cytofix/cytoperm buffer (Cat.# 554722) for 24 h at 4 °C. They were then washed twice in 1X BD Bioscience Perm/Wash (Cat.# 554723) and incubated with anti-PDX1 primary antibody (1/100, R&D Systems AF2419) and anti-NKX6-1 primary antibody (1/2000, Developmental Studies Hybridoma Bank F55A10) in BD Bioscience Perm/Wash, for 1 h at room temperature. Following two washes in the BD Bioscience Perm/Wash, donkey anti goat AF488 secondary antibody (Jackson ImmunoResearch Laboratories Inc. 705-546-147) and donkey anti-mouse AF647 (Life Technology A31571) diluted 1/400 in Perm/Wash was applied, and the cells incubated again for 1 h at room temperature. Mouse, rat, and goat IgGs were used as control. Flow cytometry was carried out using the BD LSR Fortessa flow cytometer, and data were analyzed using FlowJo software. Supplementary Table 3 for list of antibodies used for flow cytometry. In addition to the anti-GP2 antibody, two additional antibodies, HPx1 and HPx2 (Novus Biologicals, Littleton, CO) raised against human exocrine pancreas^22^ were used to track GP2^+^ cells.

### Magnetic-activated cell sorting

Cells were dissociated from the monolayer using TrypLE Express (Gibco) and washed 1X in PBS/10% fetal bovine serum (FBS; Wisent Inc.) (FACS buffer) containing 10 μM Y27632 (Tocris). Live cells were incubated for 20 min at room temperature with an anti-GP2 antibody in FACS buffer. After washing once with FACS buffer, the live cells were incubated for an additional 20 min at room temperature with an anti-mouse PE-conjugated secondary antibody in FACS buffer. The cells were washed once again with FACS buffer, and re-suspended in MACS buffer (Miltenyi Biotec) with anti-PE microbeads (Miltenyi Biotec) at a concentration of 75 μl MACS buffer and 20 μl beads/1 × 10^7^ cells. They were incubated at 4 °C for 15 min, followed by one wash in MACS buffer. The cells were then loaded onto a LS positive selection column (Miltenyi Biotec) and the flow through collected. The column was washed thrice and the cells eluted, all in FACS buffer with 10 μM Y27632 (Tocris). The elution from the LS positive selection column formed the GP2^+^ fraction. The flow through from the LS positive selection column was then loaded onto a LD depletion column (Miltenyi Biotec). The flow through from the LD depletion column was used as the flow through fraction. Following MACS, the cells were cultured as aggregates. Supplementary Table 3 for list of antibodies used.

### Quantitative PCR

The Ambion PureLink RNA mini kit was used to extract total cellular RNA. cDNA reverse transcription was then conducted using Superscript III reverse transcriptase and RNAseOUT recombinant ribonuclease inhibitor (Invitrogen). qPCR was performed using BioRad SsoAdvanced SYBR green supermix and the Biorad CFX Connect real-time system. Relative gene expression was normalized to the housekeeping gene TBP, and fold-change calculated based on a comparison to the expression level of the adult pancreas. Adult Pancreas total RNA was purchased from Takara (Cat #636577, Lot # 1202351 A). Primer sequences are listed on Supplementary Table 5.

### Immunostaining

For live staining of GP2, monolayer cultures were dissociated to single cells using TrypLE Express (Gibco) at 37 °C, and stained as described above for flow cytometry. The labeled cells were then re-suspended at a concentration of 100,000 cells/ml in 1X PBS/10% FBS (Wisent Inc.). A volume of 100 μl of cell suspension was used per slide, and centrifuged at 85 RCF in the Thermo Fisher Scientific Shandon Cytospin 4. The slides were then fixed in 1.6% PFA for 24 h at 4 °C.

hESC-derived, GP2-sorted aggregates were harvested at day 23 of differentiation and fixed in 1.6% PFA for 48 h at 4 °C. They were then embedded in agarose and paraffin, and 3 μm sections were cut by the Toronto General Hospital, Pathology Research Program Laboratory. Sections were de-paraffinized using xylene, and rehydrated in a serial dilution of absolute alcohol. Antigen-retrieval was performed, and the sections were blocked using 10% non-immune donkey serum (Jackson ImmunoResearch Laboratories Inc.) in PBS. Primary antibodies were diluted in 1X PBS supplemented with 0.3% Triton X-100 (Sigma) and 0.25% BSA (Sigma) (PBS-Triton-BSA), and incubation was conducted at 4 °C overnight. After washing, the sections were incubated with secondary antibodies in PBS-Triton-BSA for 45 min at room temperature.

Cryosections of human pancreatic tissue obtained through the Neonatal Donor Program of the International Institute for the Advancement of Medicine (IIAM) were post-fixed with 1% PFA for 10 min and permeabilized with 0.5% Triton X-100/1X PBS for 15 min at room temperature. The sections were then blocked with 5% normal donkey serum (Sigma)/1X PBS and incubated overnight at 4 °C with primary antibodies diluted in 1X PBS supplemented with 0.2% Triton X-100 and 1% BSA (PBS-Triton-BSA) as previously reported^36^. After washing, the sections were incubated with secondary antibodies in PBS-Triton-BSA for 2 h at room temperature.

Slides were counterstained with DAPI (Biotium) for 1 min, and mounted with Dako fluorescent mounting media. Digital images were acquired using the Zeiss LSM700 and LSM510 META laser scanning confocal microscopes and Zen confocal software, using ×20, ×40, and ×63 objectives. Digital images (Supplementary Fig. 2e) were acquired using a Leica DMI6000B fluorescence microscope equipped with a Leica DFC360FX digital camera. Digital images (Fig. 7) were acquired using the EVOS FL Cell Imaging System (Thermo Fisher), using ×40. Supplementary Table 4 for list of antibodies used for immunostaining.

### Glucose-stimulated insulin secretion

Six replicates of 20–25 representative H1-derived presort-derived and GP-2^+^-derived aggregates originating from three different batches of cells and collected at day 22, 24, and 25 were hand-picked and equilibrated with Krebs Ringer Buffer (KRB) + 2.8 mM glucose for 30 min, prior to sequential incubation in 100 μl low glucose KRB (2.8 mM), high-glucose KRB (16.7 mM), depolarizing KRB (45 mM KCl) for 45 min each. All incubation steps were performed at 37 °C in a tissue culture incubator. Collected supernatants were stored at 4 °C prior to use. Remaining insulin content was collected from ES cell cluster lysate by acid-ethanol extraction overnight. Insulin measurements were performed in quadruplicate by HTRF assay (Cisbio).

### Statistical analysis

All flow cytometry, and qPCR data were analyzed using an unpaired Student’s *t*-test. One-way ANOVA followed by Tukey’s multiple comparisons test was used to analyze the percentage of C-Peptide^+^/NKX6-1^+^ cells generated using H1, H9, and BJ-iPSC1 cells (Figs. 6c, 8b and Supplementary Fig. 7d) and to analyze the percentage of GP2^+^/PDX1^+^ and GP2^+^/NKX6-1^+^ generated at day 13 (Fig. 4e).

### Data availability

The authors declare that all data supporting the findings of this study are available within the article and its supplementary information files or from the corresponding author upon reasonable request. The associated raw mass spectrometry data have been deposited in a public repository (the Mass Spectrometry Interactive Virtual Environment; http://massive.ucsd.edu) under identifier MSV000081178 or directly at the link: “ftp://massive.ucsd.edu/MSV000081178”.

## Electronic supplementary material

Supplementary Information

Supplementary Data 1

Supplementary Data 2

Supplementary Data 3

Supplementary Data 4

Supplementary Data 5

Supplementary Data 6

Supplementary Data 7

Supplementary Data 8

Supplementary Data 9

Supplementary Data 10

Supplementary Data 11
