# Supplementary material for: Glycoprotein 2 is a specific cell surface marker of human pancreatic progenitors
Source: Nat Commun. 2017 Aug 24;8:331. doi: 10.1038/s41467-017-00561-0 (PMC5569081; doi:10.1038/s41467-017-00561-0)
Supplement: Supplementary file 1 — Supplementary Information [file 41467_2017_561_MOESM1_ESM.pdf]

Description of Supplementary Files

File Name: Supplementary Information
Description: Supplementary Figures, Supplementary Tables.

File Name: Supplementary Data 1
Description: 1043 protein groups identified by mass spectrometry

File Name: Supplementary Data 2
Description: Protein counts for Gene Ontology Molecular function terms

File Name: Supplementary Data 3
Description: Subset of proteins annotated for receptor activity, queried for signalling pathway involvement

File Name: Supplementary Data 4
Description: Distribution of N-linked glyco-proteins among each cell population

File Name: Supplementary Data 5
Description: Cluster 1, 126 proteins out of 184 peptides identified

File Name: Supplementary Data 6
Description: Cluster 2, 84 proteins out of 109 peptides identified

File Name: Supplementary Data 7
Description: Cluster 3, 88 proteins out of 123 peptides identified

File Name: Supplementary Data 8
Description: Cluster 4, 76 proteins out of 99 peptides identified

File Name: Supplementary Data 9
Description: Cluster 5, 226 proteins out of 308 peptides identified

File Name: Supplementary Data 10
Description: Cluster 6, 64 proteins out of 66 peptides identified

File Name: Supplementary Data 11
Description: UniProt mapping of MPC-specific genes to their translated proteins, including specification of N-glycosylated state, subcellular localization, and presence in clusters 2, 4 or 5

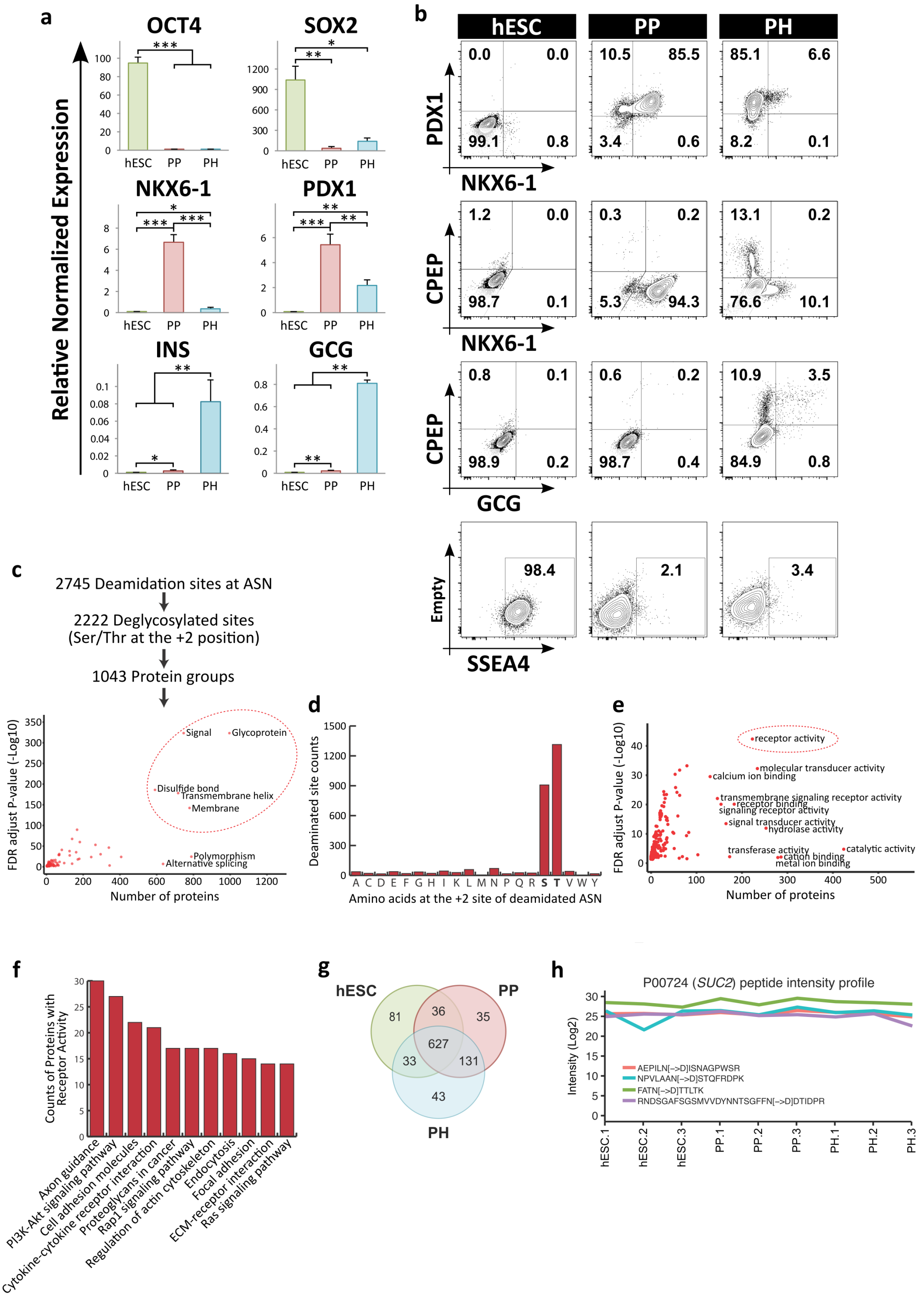

Supplementary Figure 1 Characterization of cell populations for proteomics analysis

(a) QPCR analyses of *OCT4, SOX2, NKX6-1, PDX1, INS* and *GCG* in undifferentiated hESCs (H1), and day 13 PP and PH cultures. Expression levels normalized to *TBP*, and relative to adult pancreas (equal to 1, not shown). N=3 for hESC and N=4 for PP and PH, error bars indicate s.e.m. *p<0.05, **p<0.01, ***p<0.001. (b) Flow cytometry analyses of undifferentiated hESCs (H1), and day 13 PP and PH cultures. Cells were

stained with either: anti-PDX1 in-conjunction with NKX6-1, anti-C-PEPTIDE (CPEP) in-conjunction with anti-NKX6-1, anti-CPEPTIDE in-conjunction with anti-GLUCAGON (GCG) and anti-SSEA4. (c) Scatter plot of protein keyword analysis. (d) Bar chart showing amino acids present at the +2 position of the deamiad asparagine. (e) Scatter plot of Gene Ontology Molecular function terms. (f) Bar chart showing counts of proteins with receptor activity. (g) Venn diagram depicting the distribution of N-linked glycosylated proteins identified in each of the 3 cell types. Of the 1043 proteins identified only those occurring in at least 2 out of 3 replicates are shown. (h) Intensity profile of four deglycosylation sites of P00724 (*Suc2*) yeast protein.

Abbreviations: OCT4, octamer-binding transcription factor 4; SOX2, SRY (sex determining region Y)-box 2; NKX6-1, NK6 homeobox 1; PDX1, pancreatic and duodenal homeobox 1; INS, Insulin; GCG, glucagon; CPEP, c-peptide; SSEA4, stage-specific embryonic antigen-4; ASN, asparagine; SUC2, sucrose transport protein.

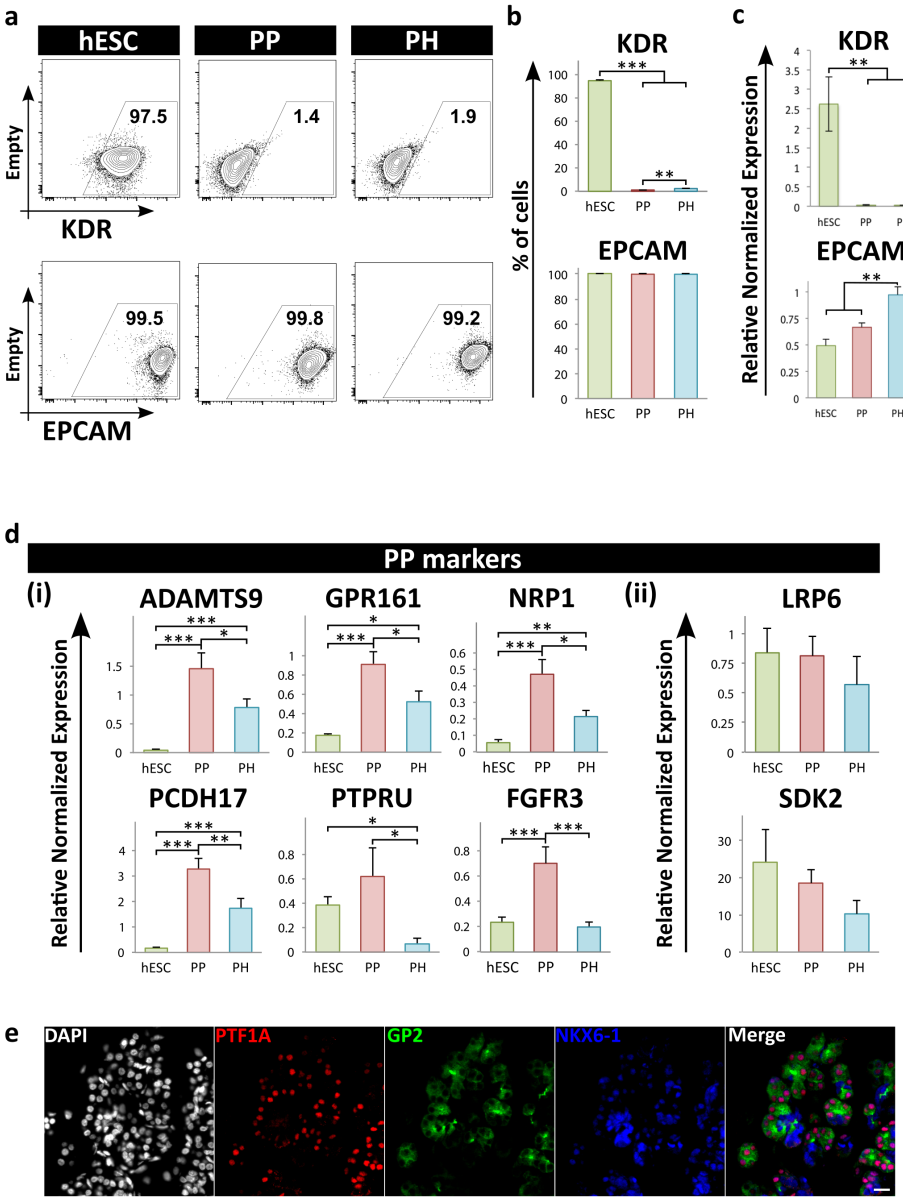

Supplementary Figure 2 Proteomics validation

(a-b) Flow cytometry analyses of undifferentiated hESCs (H1), and day 13 PP and PH cultures. Cells were stained with anti-KDR or anti-EPCAM. N=3 for hESC and N=4 for PP and PH (KDR), N=4 for hESC and N=5 for PP and PH (EPCAM) error bars indicate s.e.m. **p<0.01, ***p<0.001. (c) qPCR analysis of *KDR* and *EPCAM* N=3 for hESC and N=4 for PP and PH error bars indicate s.e.m. **p<0.01 (d) qPCR analysis of *ADAMTS9* and *GPR161* (N=6 for hESC and PH and N=5 for PP), *NRP1* (N=7 for hESC and N=8 for

PP and PH), *PCDH17* (N=5), *FGFR3* (N=10 for hESC and N=11 for PP and N=12 for PH), *PTPRU, LRP6* and *SDK2* (N=3 for hESC and N=4 for PP and PH). (i) validated markers, (ii) invalidated markers. Expression levels normalized to *TBP* and relative to adult pancreas (equal to 1, not shown). Error bars indicate s.e.m. *$p<0.05$, **$p<0.01$, ***$p<0.001$. (e) PTF1A/GP2/NKX6-1 immunostaining of human pancreas at gestational week 37. DAPI was used to counterstain nuclei. Scale bar represents 20 µm.
Abbreviations: KDR, kinase insert domain receptor; EPCAM, epithelial cell adhesion molecule; ADAMTS9, a disintegrin and metalloproteinase with thrombospondin motifs 9; GPR161, G-protein coupled receptor 161; NRP1, neuropilin 1; PCDH17, protocadherin-17; PTPRU, receptor-type tyrosine-protein phosphatase U; FGFR3, fibroblast growth factor receptor 3; LRP6, low-density lipoprotein receptor-related protein 6; SDK2, protein sidekick-2.

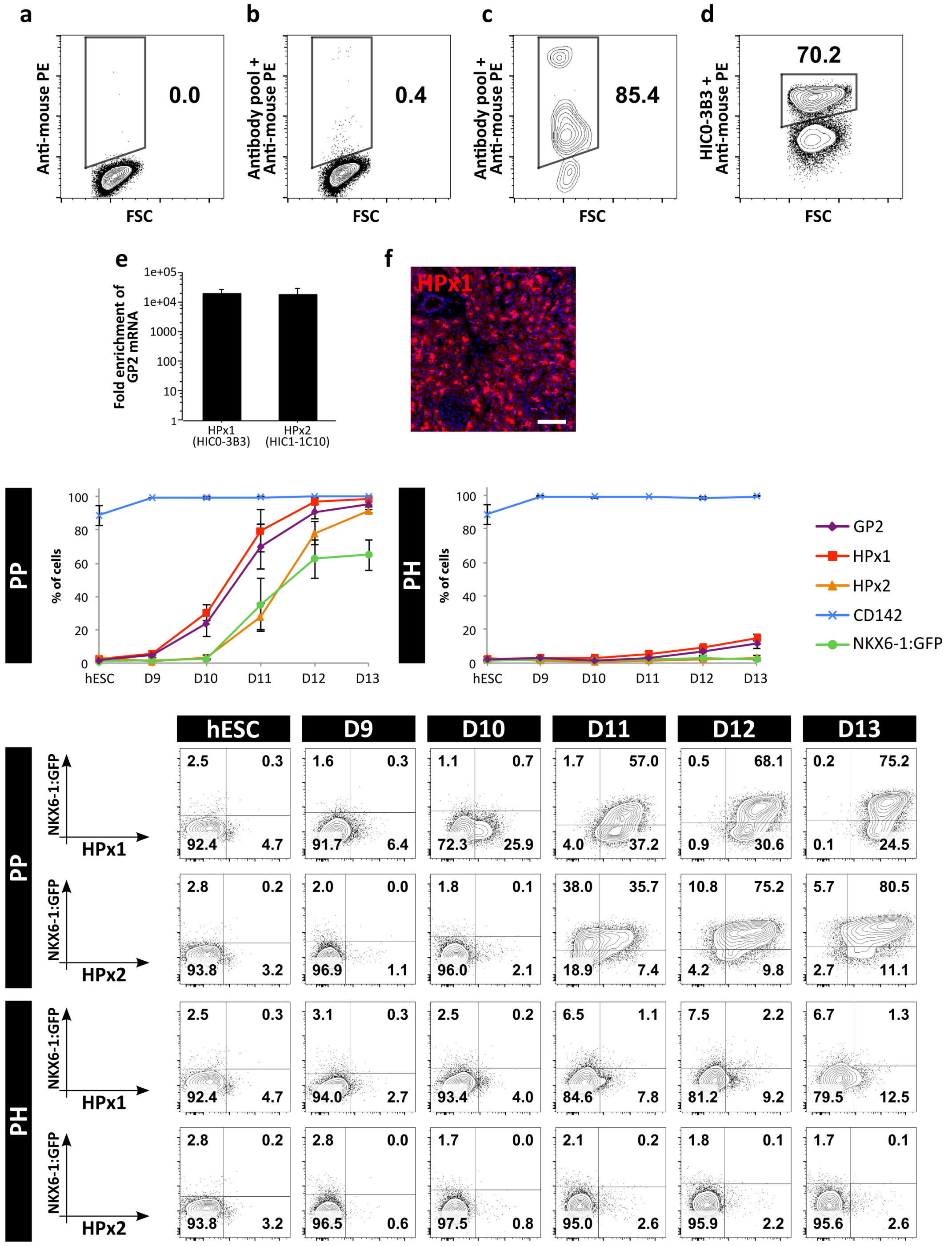

Supplementary Figure 3 HPx1 and HPx2 can be used to label NKX6-1-GFP+ cells

The antigens for HPx1/HIC0-3B3 and HPx2/HIC1-1C10 were identified by viral cDNA library screening. C6 rat glioma cells were transduced with retrovirus containing a human pancreas cDNA library using the Stratagene Viraport system. FACS was then used to identify and recover cells positive for any of a pool of 10 antibodies including HPx1 and HPx2. Unlabeled cells are shown in (a), and labeled cells pre- (b) and post- (c) sort are also indicated. Following a week of cell culture to increase cell numbers,

cells were labeled with individual antibodies and re-sorted to collect those with specific reactivity against HPx1 (d) for DNA recovery, viral DNA insert sequencing, and BLAST query. (e) Enrichment of GP2 mRNA in FACS sorted $HPx1^{+}$ and $HPx2^{+}$ cells recovered from human pancreatic tissue compared to cells negative for these markers. (f) The labeling patterns of HPx1 on cryosections of human pancreas. The illustrated 5 µm cryosection was labeled with HPx1 and detected with Cy3-conjugated anti-mouse IgG, scale bar represents 25 µm. (g-h) Flow cytometry analysis of undifferentiated NKX6-$1^{GFP/w}$ hESCs, PP and PH cultures from day 9 to 13 of differentiation. Cells were stained with anti-HPx1, or anti-HPx2. N=3, error bars indicate s.e.m. The graph lines for CD142, NKX6-1:GFP and GP2 are from the same experiments as presented in Figure 3, the cells from three of those experiments were co-stained with HPx1 and HPx2 as well as CD142 and GP2.

Abbreviations: FSC, forward scatter; HPx1, human pan-exocrine antibody 1; D9, day9; GP2, pancreatic secretory granule membrane major glycoprotein 2; HPx2, human pan-exocrine antibody 2; CD142, tissue factor; NKX6-1, NK6 homeobox 1.

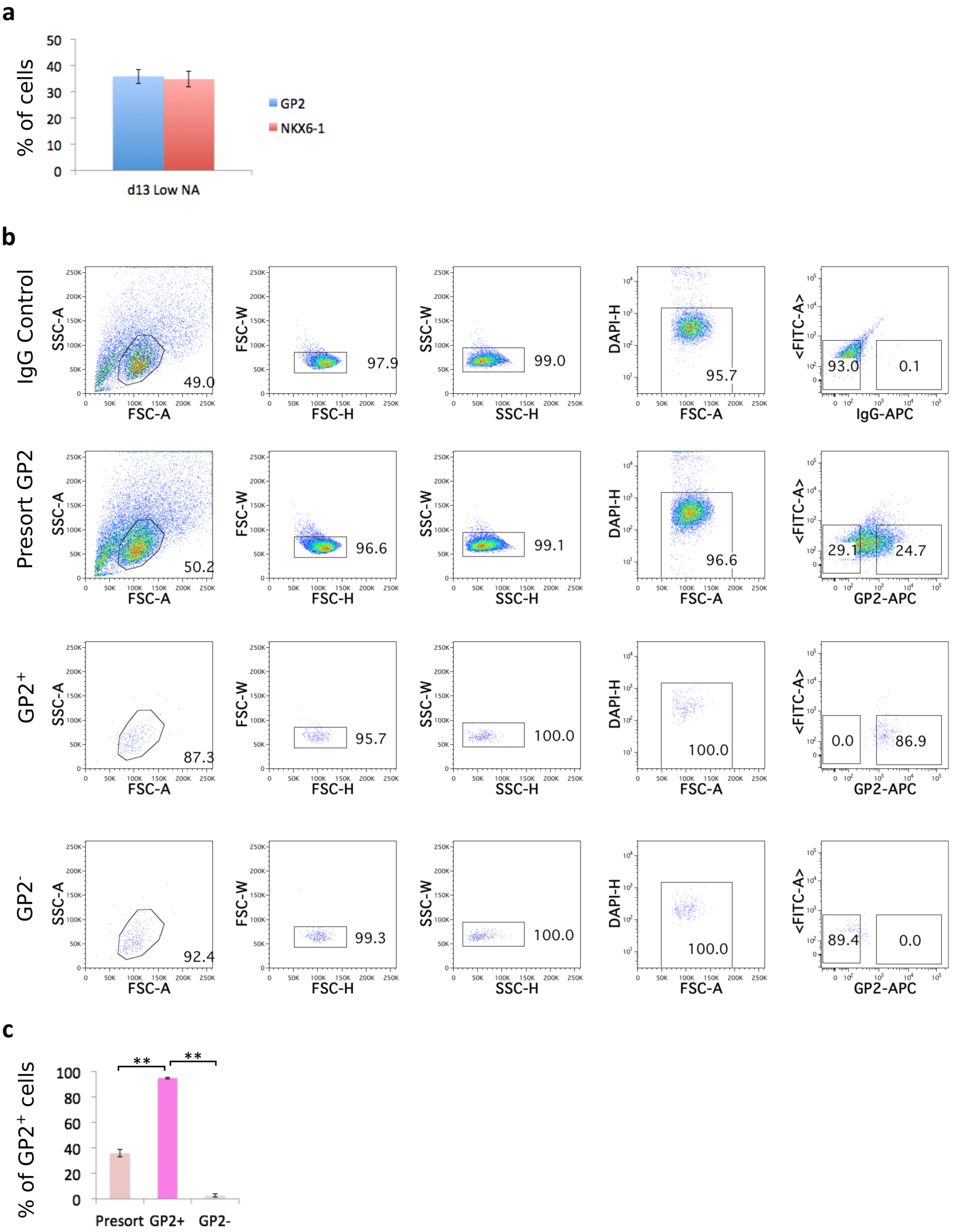

Supplementary Figure 4 GP2 sorting strategy

(a) Average percentage of GP2 and NKX6-1+ cells as measured by flow cytometry at day 13 of differentiation using H1 hESC line N=5, error bars indicate s.e.m. (b) Flow cytometry representation of the FAC-sorting strategy; GP2+ and GP2- cells were obtained following H1 differentiation with 3.3mM nicotinamide at stage 4. Gates are placed based on forward and side scatter profiles, doublet and DAPI+ exclusion. IgG control is used to set the negative and positive gates. Purity check is presented for

GP2+ and GP2- sorted cells. (c) Average percentage of GP2+ cells obtained post-sort. N=5, error bars indicate s.e.m. **p<0.01.

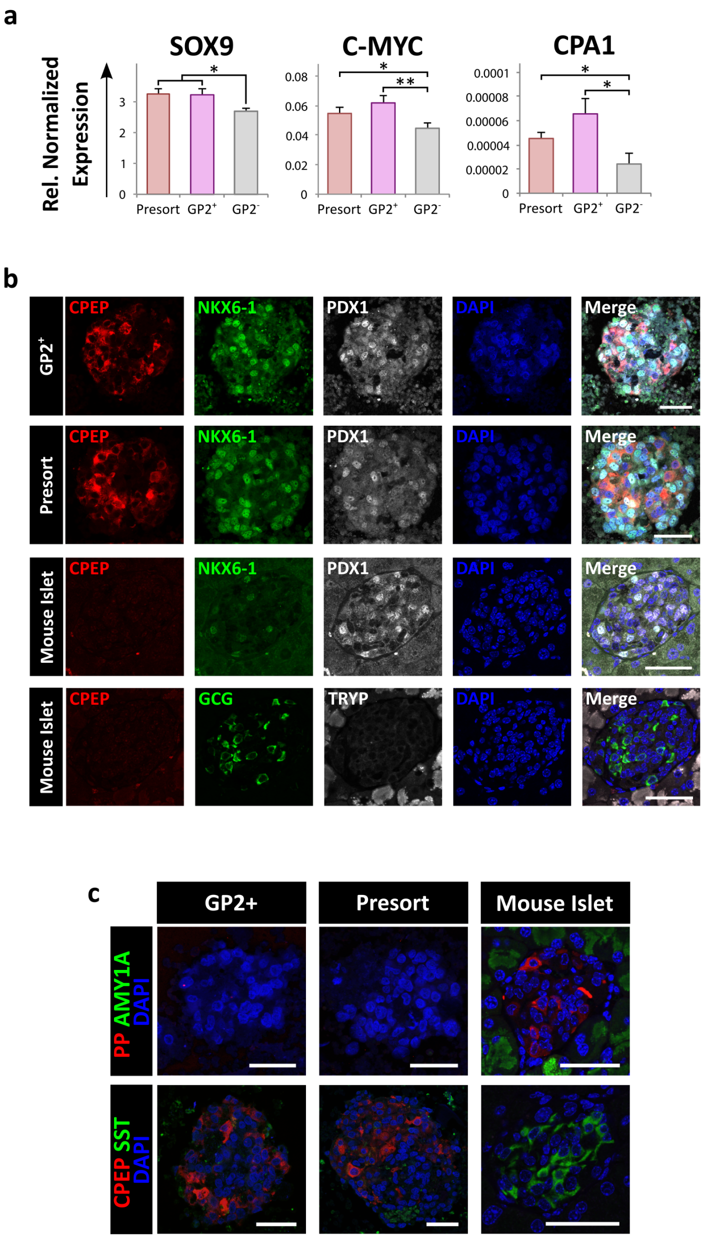

Supplementary Figure 5 Characterization of GP2 sorted cells and developing aggregates

(a) qPCR analysis of *SOX9* (N=4), *C-MYC* (N=5) and *CPA1* (N=3). Expression levels normalized to *TBP*, and relative to adult pancreas (equal to 1, not shown). Error bars indicate s.e.m. *p<0.05, **p<0.01. (b) C-PEPTIDE (C-PEP)/NKX6-1/PDX1 and C-PEPTIDE (CPEP)/GLUCAGON (GCG)/TRYPSIN (TRYP) immunostaining of GP2+ and unsorted (PRESORT) H1 cells at day 23 of differentiation and mouse islets as control,

note that anti-c-peptide antibody is human-specific. Scale bar represents 50 µm. (c) PANCREATIC POLYPEPTIDE (PP)/AMYLASE (AMY1A) and C-PEPTIDE/SOMATOSTATIN (SST) immunostaining of $GP2^{+}$ and presort H1 cultures at day 23 of differentiation, and mouse islets. Scale bar represents 50 µm.

Abbreviations: SOX9, SRY (sex determining region Y)-box 9; C-MYC, v-myc avian myelocytomatosis viral oncogene homolog; CPA1, carboxypeptidase A1; GP2, pancreatic secretory granule membrane major glycoprotein 2; NKX6-1, NK6 homeobox 1; PDX1, pancreatic and duodenal homeobox 1; CPEP, c-peptide; GCG, glucagon; TRYP, trypsin; PP, pancreatic polypeptide; AMY1A, amylase alpha 1A; SST, somatostatin.

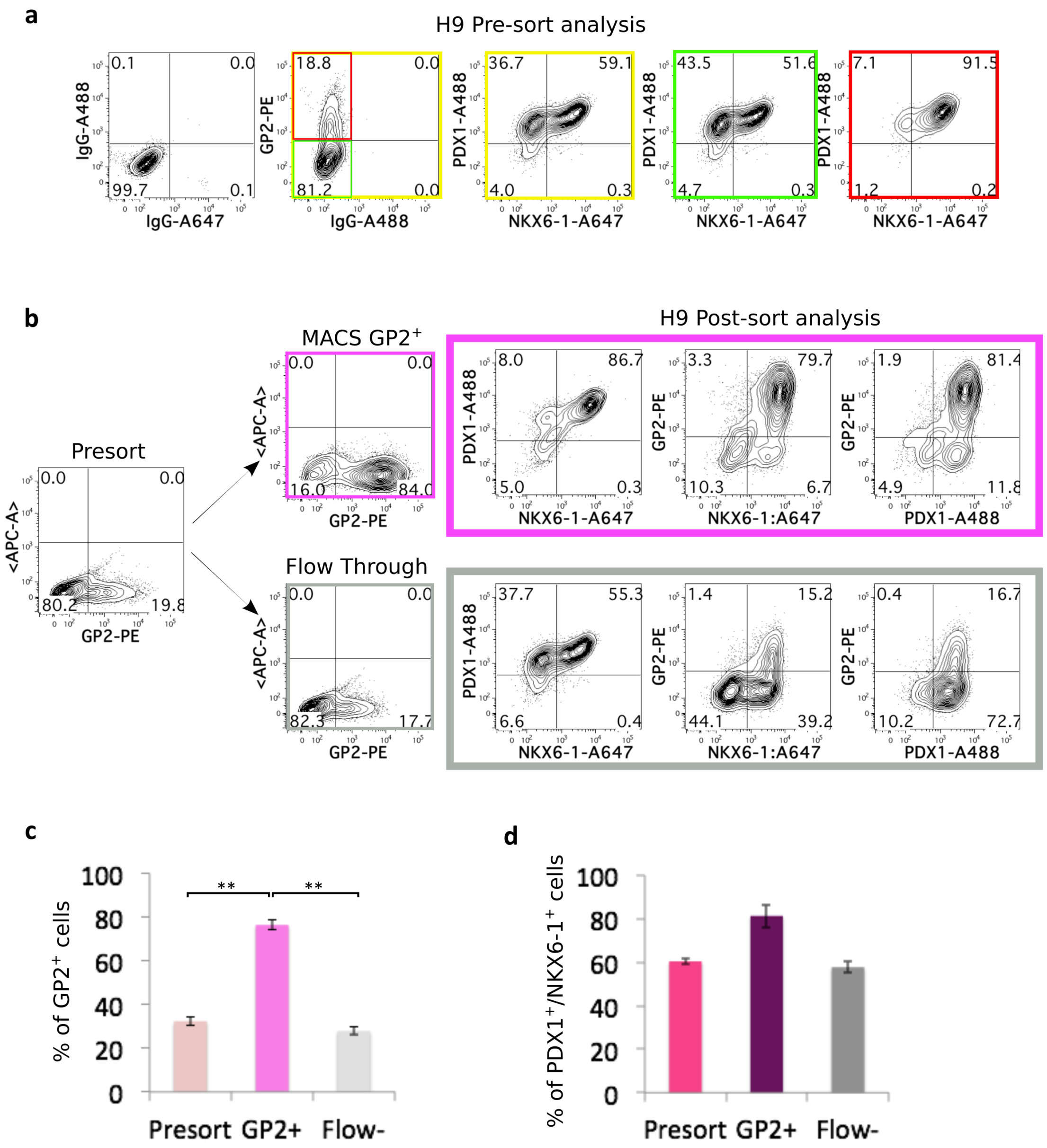

Supplementary Figure 6 H9 pre- and post-sort characterization

(a) Flow plots showing the GP2, PDX1 and NKX6-1 profiles at day 13 of the unsorted (presort) H9 cells. Note that the GP2+ fraction (red box) is almost exclusively PDX+/NKX6-1+. (b) Flow plots showing the GP2 profile at day 13 of differentiation of H9 cells. Cells were analyzed either before MACS sorting (presort), after GP2 enrichment from a positive selection column (GP2^{+}) or in the flow through from a depletion column. Cells were stained with an anti-GP2 primary antibody, anti-mouse

(PE-conjugated) secondary antibody, and anti-PE magnetic beads. H9 post-sort analysis shows NKX6-1, PDX1 and GP2 expression by flow cytometry in day 13 $GP2^{+}$ sorted and Flow through H9 cells. (c) The bar graph shows the average percentage of $GP2^{+}$ cells obtained at day 13 in presort, MAC-sorted GP2+ and Flow through fraction (Flow-). N = 7, error bars indicate s.e.m. **p<0.01. (d) The bar graph shows the average percentage of PDX+/NKX6-1+ cells obtained at day 13 in presort, MAC-sorted GP2+ and Flow through fraction (Flow-). N = 2, error bars indicate average deviation.

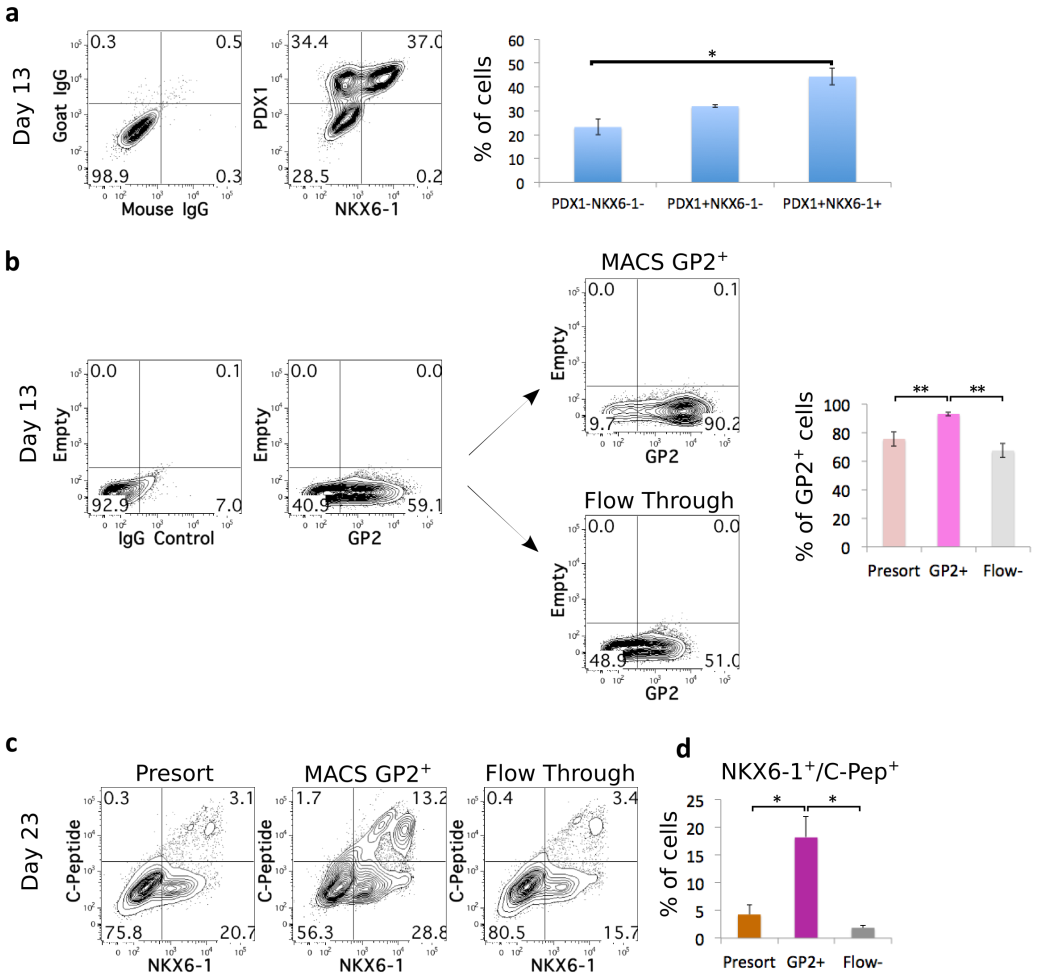

Supplementary Figure 7 BJ-iPSC1 pre and post-sort characterization

(a) Flow plots showing a representative PDX1 and NKX6-1 profile at day 13 of the unsorted (presort) BJ-IPSC1 cells and quantification of the PDX1$^-$/NKX6-1$^-$, PDX1$^-$/NKX6-1$^+$ and PDX1$^+$/NKX6-1$^+$fractions, N=5 error bars indicate s.e.m. *p<0.05. (b) Flow plots showing the GP2 profile at day 13 of differentiation of BJ-IPSC1 cells. Cells were analyzed either before MACS sorting (presort), after GP2 enrichment from a

positive selection column ($GP2^+$) or in the flow through from a depletion column. Cells were stained with an anti-GP2 primary antibody, anti-mouse (PE-conjugated) secondary antibody, and anti-PE magnetic beads. The bar graph shows the average percentage of $GP2^+$ cells at day 13 in Presort, GP2+ and Flow through (Flow-) fractions. N = 5, error bars indicate s.e.m. **$p<0.01$. (c) Following MACS sorting for GP2 at day 13, cells were then cultured to generate β-like cells up to day 23. Representative flow cytometry plots of NKX6-1 and C-PEPTIDE (CPEP) expression at day 23 of differentiation from either unsorted (PRESORT), enriched for GP2 using a MACS positive selection column ($GP2^+$) or in the flow through cell population. The bar graph shows the average percentage of double positive NKX6-1^+/C-$PEPTIDE^+$ cells at Day 23. N = 4, error bars indicate s.e.m. *$p<0.05$.

Supplementary Table 1: Proteins from cluster 1 that were previously identified in PSC-restricted cells

Protein Name	Gene
Angiopoietin-1 receptor	TEK
Anosmin-1	KAL1
Carbohydrate sulfotransferase 6	CHST6
Contactin-1	CNTN1
Ectonucleoside triphosphate diphosphohydrolase 2	ENTPD2
Gamma-aminobutyric acid receptor subunit alpha-3	GABRA3
Gamma-aminobutyric acid receptor subunit alpha-5	GABRA5
Interleukin-17 receptor D	IL17RD
Leucine-rich repeat and immunoglobulin-like domain-containing nogo receptor-interacting protein 1	LINGO1
N-acetylglucosamine-1-phosphotransferase subunits alpha/beta;N-acetylglucosamine-1-phosphotransferase subunit alpha;N-acetylglucosamine-1-phosphotransferase subunit beta	GNPTAB
N-acetyllactosaminide beta-1,6-N-acetylglucosaminyl-transferase, isoform B	GCNT2
Netrin receptor UNC5D	UNC5D
Neurexin-1	NRXN1
Plexin domain-containing protein 2	PLXDC2
Receptor-type tyrosine-protein phosphatase zeta	PTPRZ1
Semaphorin-6A	SEMA6A
Sodium/potassium-transporting ATPase subunit beta-2	ATP1B2
Tumor necrosis factor receptor superfamily member 11A	TNFRSF11A
UDP-GlcNAc:betaGal beta-1,3-N-acetylglucosaminyltransferase 2	B3GNT2
V-set and immunoglobulin domain-containing protein 10	VSIG10
Vascular endothelial growth factor receptor 1	FLT1
Vascular endothelial growth factor receptor 2	KDR
Versican core protein	VCAN

Supplementary Table 2: List of antibodies selected as novel markers of PPs

Protein Name	Gene	Cluster
A disintegrin and metalloproteinase with thrombospondin motifs 9	ADAMTS9	2
Fibroblast growth factor receptor 3	FGFR3	2
G-protein coupled receptor 161	GPR161	2
Low-density lipoprotein receptor-related protein 6	LRP6	2
Neuropilin-1	NRP1	5
Pancreatic secretory granule membrane major glycoprotein 2	GP2	2 & 5
Protein sidekick-2	SDK2	2
Protocadherin-17	PCDH17	2
Receptor-type tyrosine-protein phosphatase U	PTPRU	2

Supplementary Table 3: List of antibodies for flow cytometry, FACS and MACS

Ab	Antigen/ Fluorophore	Company	Dilution
1°	CD142_PE	BD Bioscience (550312)	1/10
1°	CD90_APC	BD Pharmingen (559869)	1/1000
1°	CPEP	Developmental Studies Hybridoma Bank, University of Iowa (GN-ID4)	1/1000
1°	EPCAM_PE	eBioscience (12-9326-42)	1/20
1°	FGFR3	GeneTex (GTX64093)	1/10
1°	GCG	Sigma-Aldrich (G2654)	1/2000
1°	GP2	MBL International Corporation (D277-3)	1/10,000
1°	HPx1	Craig Dorrell, Oregon Health & Science University (HIC0-3B3)	1/20
1°	HPx2	Craig Dorrell, Oregon Health & Science University (HIC1-1C10)	1/20
1°	KDR_PE	R&D Systems (FAB357P)	1/7
1°	NKX6-1	Developmental Studies Hybridoma Bank, University of Iowa (F55A10)	1/2000
1°	NRP1	Thermo Scientific (PA5-26079)	1/10
1°	PDX1	R&D Systems (AF2419)	1/100
1°	SSEA4_PE	BD Pharmingen (560128)	1/100
2°	AF488	Life Technologies (A21202)	1/400
2°	AF488	Jackson ImmunoResearch Laboratories Inc. (705-546-147)	1/400
2°	AF647	Life Technologies (A31571)	1/400
2°	AF647	Life Technologies (A31573)	1/400
2°	AF647	Jackson ImmunoResearch Laboratories Inc. (705-606-147)	1/400
2°	PE	BD Pharmingen (550767)	1/400
2°	PE	Jackson ImmunoResearch Laboratories Inc. (115-115-164)	1/800
Isotype control	Mouse IgG1	Biolegend (400102)	
Isotype control	Rat IgG2a	Life Technologies (R2A00)	
Isotype control	Rabbit IgG	Santa Cruz Biotechnology (sc-2027)	
Isotype control	Mouse IgG	Jackson ImmunoResearch Laboratories Inc. (015-000-003)	
Isotype control	Goat IgG	R&D Systems (AB-108-C)	

Supplementary Table 4: List of antibodies for immunostaining

Ab	Antigen/ Fluorophore	Company	Dilution
1°	AMY1A	Sigma-Aldrich (WH0000276M4)	1/500
1°	CPEP	Developmental Studies Hybridoma Bank, University of Iowa (GN-ID4)	1/1000
1°	GCG	Sigma-Aldrich (G2654)	1/500
1°	GP2	MBL International Corporation (D277-3)	1/250
1°	HPx1	Craig Dorrell, Oregon Health & Science University (HIC0-3B3)	1/20
1°	INS	Dako (A0564)	1/1000
1°	NKX6-1	Developmental Studies Hybridoma Bank, University of Iowa (F55A10)	1/2000
1°	NKX6-1	Palle Serup, Beta Cell Biology Consortium	1/2000
1°	PDX1	Abcam (ab47383)	1/10,000
1°	PP	Peninsula Laboratories LLC (T-4088)	1/250
1°	PTF1A	Christopher Wright, Vanderbilt University	1/500
1°	SST	Thermo Scientific (PA1-30636)	1/500
1°	TRYP	R&D Systems (AF3586)	1/300
1°	NKX2-2	Developmental Studies Hybridoma Bank, University of Iowa (74.5A5)	1/00
1°	MAFA	Kind gift from Dr. Alireza Rezania (Custom Ab Lifespan Biosciences, Seattle WA)	1/100
1°	GLUT1	Thermo Scientific (PA5-16793)	1/200
2°	AF488	Life Technologies (A21202)	1/800
2°	AF488	Jackson ImmunResearch Laboratories Inc. (706-545-148)	1/800
2°	AF647	Jackson ImmunoResearch Laboratories Inc. (705-606-147)	1/800
2°	AF647	Jackson ImmunoResearch Laboratories Inc. (313-605-003)	1/800
2°	AF647	Life Technologies (A31571)	1/800
2°	AF647	Life Technologies (A31573)	1/800
2°	Cy2	Jackson ImmunResearch Laboratories Inc. (715-225-150)	1/500
2°	Cy2	Jackson ImmunResearch Laboratories Inc. (711-225-152)	1/500
2°	Cy3	Jackson ImmunResearch Laboratories Inc. (711-165-152)	1/500
2°	Cy3	Jackson ImmunResearch Laboratories Inc. (705-165-147)	1/500
2°	Cy3	Jackson ImmunoResearch Laboratories Inc. (712-165-153)	1/400
2°	Cy5	Jackson ImmunResearch Laboratories Inc. (706-175-178)	1/200
2°	Cy5	Jackson ImmunResearch Laboratories Inc. (715-175-150)	1/200
2°	DyLight 550	Thermo Scientific (SA5-100)	1/400

Supplementary Table 5: Primer list

Primer	Sequence
ADAMTS9 F	GAA CGC GAC GGA GCA TTA AC
ADAMTS9 R	GAC AGT GAA CAG TGG AGC GA
CD142 F	CAG AGT GTG ACC TCA CCG AC
CD142 R	GAA CCG GTG CTC TCC ACA TT
CPA1 F	GCA TCC AGG CGG TCA AGA T
CPA1 R	GAC TGC ACG TCC TCG ATC AT
EPCAM F	GGG CCC TCC AGA ACA ATG AT
EPCAM R	AGT GTT CAC ACA CCA GCA CA
FOXA2 F	GCA TTC CCA ATC TTG ACA CGG TGA
FOXA2 R	GCC CTT GCA GCC AGA ATA CAC ATT
GCG F	AAG CAT TTA CTT TGT GGC TGG ATT
GCG R	TGA TCT GGA TTT CTC CTC TGT GTC T
GP2 F	AAC CCT TCC GAA GCA CAG AG
GP2 R	GGA CAC AGG TCT CCG ACA TC
GPR161 F	GAA GAT CAC AGG GAA CCG GG
GPR161 R	CTC CAC GGA TGA CCA ACC AA
INS F	AGA AGC GTG GCA TTG TGG AAC A
INS R	TAT TCC ATC TCT CTC GGT GCA GGA
KDR F	CGT GTC TTT GTG GTG CAC TG
KDR R	AAC AGG TGT GGG CAA CTC TC
LRP6 F	ACA GAC ACT GGC ACT GAT CG
LRP6 R	ATA GCC CGG GGT TCC TCT AA
MYC F	CAT ACA TCC TGT CCG TCC AAG
MYC R	GAG TTC CGT AGC TGT TCA AGT
NGN3 F	GCG CAA TCG AAT GCA CAA CCT CAA
NGN3 R	TTC GAG TCA GCG CCA AGA TGT AGT T
NKX6.1 F	AGA GGA CGA CGA CTA CAA TAA GCC
NKX6.1 R	ACT TGT GCT TCT TCA ACA GCT GCG
NRP1 F	ATG CCT GAA AAC ATC CGC CT
NRP1 R	GTG CTT CCC ACC CTG AAT GA
OCT4 F	ATG CAT TCA AAC TGA GGT GCC TGC
OCT4 R	CCA CCC TTT GTG TTC CCA ATT CCT
PCDH17 F	GCA GTC AGT ACT TGC CCA CT
PCDH17 R	GAA GCC ATG AAG GGA GGG TC
PDX1 F	TAC TGG ATT GGC GTT GTT TGT GGC
PDX1 R	AGG GAG CCT TCC AAT GTG TAT GGT
PTF1A F	TTA TCC GAA CAG CCA AAG TCT GGA CC
PTF1A R	AGT CTG GGA CCT CTC AGG ACA CAA
PTPRU F	CAG CCG CTA CAC CAT CAA GA
PTPRU R	CCC CTC AGG GTT AGT GAG GA
SDK2 F	CCT GAG TCC GTG GGC TAT AA
SDK2 R	TCC TCG ATG GTG TAG TCC CG
SOX17 F	AGG AAA TCC TCA GAC TCC TGG GTT
SOX17 R	CCC AAA CTG TTC AAG TGG CAG ACA
SOX2 F	GGA TAA GTA CAC GCT GCC CG
SOX2 R	ATG TGC GCG TAA CTG TCC AT
SOX9 F	TGC ATT TCC TCC TGC CTT TGC TTG
SOX9 R	GGG CAC TTA TTG GCT GCT GAA ACA
TBP F	TGA GTT GCT CAT ACC GTG CTG CTA
TBP R	CCC TCA AAC CAA CTT GTC AAC AGC
